# From preclinical promise to regulatory reality: translation of small rodent acute injury therapies to human approval and safety outcomes: A systematic review

**DOI:** 10.1371/journal.pone.0358773

**Published:** 2026-09-28

**Authors:** Timothy R. Entwistle, William R. Cowey, Eliza Stokoe, John P. Stone, James E. Fildes

**Affiliations:** 1 Pebble Institute, Manchester, United Kingdom; 2 Pebble Biotechnology Laboratories, Manchester, United Kingdom; Lorestan University, IRAN, ISLAMIC REPUBLIC OF

## Abstract

**Background:**

Preclinical animal studies are considered essential for therapeutic development, yet translation to human benefit remains poor. The commonly cited translation rate of approximately 20% conflates trial entry with regulatory approval and may substantially overestimate the predictive validity of animal models for specific therapeutic indications.

**Objectives:**

To determine, for preclinical acute injury studies in small animal models: (a) the proportion progressing to human clinical trials; (b) the proportion achieving regulatory approval for the tested indication; (c) whether approvals represented novel translations or repurposed/class-effect approvals; and (d) post-market safety outcomes for translational successes.

**Methods:**

Systematic review following PRISMA 2020 and SYRCLE guidance. We included controlled preclinical studies in small animal models (rats, mice, guinea pigs, hamsters, rabbits) of acute injury that tested a therapeutic intervention not already in routine clinical use, published 1990–2010. MEDLINE (via PubMed) was searched on 22 March 2022; full strategy in S1 File, Section S3. Five reviewers independently screened records in duplicate. Risk of bias was assessed with the SYRCLE tool. Therapies were grouped by active ingredient and classified against pre-specified regulatory categories by two reviewers blinded to post-market safety, with a third adjudicating. Post-market safety used five pre-defined criteria. Translation rates were synthesised descriptively as proportions with 95% Wilson score confidence intervals; no meta-analysis was performed. The review was registered on OSF (https://osf.io/w97fd).

**Results:**

Of 3,847 records identified, 1,257 studies met inclusion criteria, yielding 100 distinct therapies with sufficient clinical outcome data (83 drugs, 17 devices/physical interventions). Translation-to-trial was 20.0% (251/1,257; 95% CI 17.9–22.4%). Among 83 drug therapies, only 3 (3.6%; 95% CI 1.2–10.1%) achieved first-in-class regulatory approval for the tested indication; 15 (18.1%) definitively failed in clinical trials. In an exploratory post-market analysis, 86% of drugs meeting any definition of translational success subsequently demonstrated major safety concerns; only beractant (Survanta®) remains in routine clinical use for the tested indication without major caveats, and this was not a novel first-in-class translation.

**Conclusions:**

Within acute injury research, the true regulatory success rate for novel preclinical therapies is 3.6%, likely an upper bound given publication bias. Most approved drugs subsequently experienced serious post-market safety concerns. Small rodent models have limited demonstrated predictive validity for human therapeutic outcomes in this domain.

**Registration:**

Open Science Framework, https://osf.io/w97fd.

## Background

Preclinical animal studies form the foundation of modern therapeutic development. Regulatory agencies worldwide require evidence of safety and efficacy in animal models before human trials can proceed, based on the assumption that animal physiology provides meaningful prediction of human responses [1–3]. In acute injury research, encompassing traumatic brain injury, spinal cord injury, burns, wounds, and acute organ damage, small rodent models dominate the preclinical literature due to their accessibility, short reproductive cycles, and established experimental protocols [4,5].

The translation of preclinical findings to human therapeutic benefit has been widely characterised as problematic [6–9]. Estimates suggest that only 5–10% of drugs entering clinical trials ultimately achieve regulatory approval [10–12]. However, systematic quantification of translation rates from animal studies to human outcomes remains limited, particularly for acute injury indications where the translational gap is suspected to be especially wide [13].

Previous analyses have suggested translation rates of approximately 20% from preclinical studies to clinical trials [13,14]. However, it is important to understand what these prior studies measured. Most counted any entry into human clinical testing as ‘translation’, regardless of trial outcome. They typically included drugs already approved for other indications (repurposed drugs) and did not distinguish between drugs that subsequently succeeded versus failed in trials. Furthermore, they did not follow therapies through to post-market surveillance to determine whether initial approvals were sustained by real-world safety and efficacy data.

The 20% figure may therefore be misleading for several reasons. First, it conflates translation to trial initiation with translation to regulatory approval, fundamentally different endpoints with very different implications for the value of preclinical evidence. Second, it includes repurposed drugs where the preclinical model is not truly being tested as a predictor of novel therapeutic success; the drug already had established human safety data from its original indication. Third, it does not distinguish between drugs approved for the indication tested preclinically versus those approved for entirely different conditions, which does not validate the animal model’s predictive capacity for the original therapeutic target.

Furthermore, translation analyses typically end at regulatory approval, ignoring post-market outcomes. A drug that achieves approval but subsequently causes significant harm, through adverse events not predicted by preclinical studies, represents a failure of the preclinical paradigm to predict human outcomes, regardless of initial approval status. Such post-market failures may be particularly informative about the limitations of animal models, yet they are rarely incorporated into translation metrics [15,16].

We therefore conducted a systematic review of preclinical acute injury studies in small animal models to determine: (a) the proportion progressing to human clinical trials; (b) the proportion achieving regulatory approval for the tested indication; (c) whether approved therapies represented novel translations or repurposed/class-effect drugs; and (d) the post-market safety outcomes for therapies classified as translational successes. We hypothesised that when stringent criteria are applied, the true translation rate would be substantially lower than commonly reported, and that therapies achieving approval might demonstrate significant post-market safety concerns that preclinical studies failed to predict.

## Methods

### Protocol and registration

This systematic review was conducted in accordance with the Preferred Reporting Items for Systematic Reviews and Meta-Analyses (PRISMA) 2020 guidelines [17] (S1 File, Section S1) and the SYRCLE protocol format for systematic reviews of animal intervention studies [18,19]. The review protocol was documented a priori (March 2022) and has been registered on the Open Science Framework (https://osf.io/w97fd). At the time of protocol development, PROSPERO did not routinely accept animal systematic review protocols. All methodological decisions were finalised before analysis commenced, and protocol deviations are documented transparently in S1 File, Section S2.

### Ethics statement

Not applicable. This study involved secondary analysis of published data only; no human participants or animals were directly involved. No ethical approval or participant consent was therefore required.

### Eligibility criteria

Studies were eligible for inclusion if they met the following criteria: (a) used small animal models (rats, mice, guinea pigs, hamsters, or rabbits) of acute injury; (b) tested a therapeutic intervention intended to treat or ameliorate the injury; (c) reported efficacy outcomes comparing treatment versus control groups; (d) were published between 1 January 1990 and 31 December 2010; and (e) were original research articles published in English.

Studies were excluded if they were fundamental biology without therapeutic intervention, reviews or editorials, lacked a comparator group, or tested therapies already in routine clinical use at the time of publication. The latter criterion was applied to focus the analysis on novel or experimental agents where the predictive validity of the animal model was genuinely being tested, rather than established therapies being evaluated for new applications.

The 1990–2010 timeframe was selected to allow sufficient follow-up time for translation assessment (minimum 15 years post-publication for the most recent studies). ‘Acute injury’ was operationally defined as tissue damage occurring over a short timeframe (hours to days) rather than chronic degenerative conditions. Borderline cases (e.g., certain ischaemia-reperfusion models where timing was ambiguous) were discussed by the review team and decisions documented. Examples of included injury models: traumatic brain injury, spinal cord contusion, thermal burns, acute lung injury/ARDS, radiation injury, acute kidney injury. Examples of excluded conditions: chronic neuropathic pain, progressive neurodegenerative diseases, chronic wound healing in diabetic models (unless the acute component was the explicit focus of the intervention).

### Information sources and search strategy

MEDLINE via PubMed was searched using comprehensive Boolean combinations of terms across five conceptual categories: (a) acute injury terms (acute wound, acute injury, acute trauma); (b) therapy/treatment terms (therapy, healing, care, treatment, drug, medication, regimen, management); (c) animal model terms (rat, mouse, rodent, guinea pig, hamster, rabbit); (d) human translation terms (for sensitivity checking); and (e) date restrictions (1990–2010). Multiple search set combinations (Sets 8–15) were executed to enhance sensitivity while maintaining specificity. PubMed Automatic Term Mapping was active in all searches, meaning search terms were automatically mapped to corresponding MeSH terms and entry terms. The complete search strategy with all Boolean strings is provided in S1 File, Section S3.

Database justification: PubMed/MEDLINE was the primary database. For preclinical rodent acute injury literature, PubMed provides extensive coverage as the dominant indexing service for biomedical journals publishing animal research. Previous methodological studies have shown high overlap between PubMed and other databases (EMBASE, Web of Science) for preclinical literature, particularly for rodent studies [20]. To assess the completeness of our search, we conducted reference list screening of included studies and relevant systematic reviews, which identified no additional eligible studies not already captured by our PubMed search. Additionally, a targeted sensitivity check of Embase for three exemplar therapies (progesterone, erythropoietin, minocycline) similarly identified no additional eligible preclinical studies beyond those in our dataset.

### Selection process

Study selection was conducted in two phases by five reviewers (TRE, WC, ES, JPS, JEF): title/abstract screening followed by full-text review. Each study was assessed by at least two reviewers at each phase. For each study, the lead reviewer presented the record after all reviewers had read it independently, followed by a group consensus vote. The eligibility criteria produced clear-cut classification decisions (studies were either preclinical acute injury therapy studies or they were not), and no formal disagreements required third-party adjudication across the entire screening process. An AI validation provided independent verification of the reliability of consensus decisions (see below). Studies excluded at full-text review were removed according to an exclusion hierarchy: (a) unsuccessful/negative preclinical study (no studies excluded for this reason); (b) does not meet population, intervention, or outcome criteria; (c) predatory journal or unreliable source; (d) existing clinical therapy at time of publication; (e) fundamental biology study without therapeutic application; or (f) not therapy research and development.

### AI-assisted validation of screening decisions

To provide independent verification of human screening decisions and enhance methodological rigour, AI-assisted validation was conducted using a stratified random sample of 200 studies (100 included, 100 excluded; random seed 42 for reproducibility), independently classified by a large language model (Claude, Anthropic) using identical eligibility criteria provided to human reviewers [21]. Cohen’s kappa coefficient was calculated to assess inter-rater reliability between human consensus decisions and AI classifications. Studies with discordant human-AI classifications were flagged for secondary review by senior reviewer consensus. Full AI validation methods and results are provided in S1 File, Section S4.

### Therapy grouping and classification

Multiple preclinical studies testing the same therapeutic agent were grouped by active ingredient or biological entity (not by formulation, dosing schedule, or route of administration) for the purpose of clinical/regulatory outcome mapping. For example, all studies testing ‘minocycline’ were grouped together regardless of dose, timing, or route. For therapies with mixed preclinical results (e.g., some positive and some negative studies), a classification of ‘translating’ was applied if clinical trial progression was documented in regulatory filings, ClinicalTrials.gov registrations, or peer-reviewed publications, not based on preclinical vote-counting alone. Combination therapies were classified by the novel component; studies testing combinations of two established agents were excluded.

### Risk of bias assessment

Risk of bias in included animal studies was assessed using SYRCLE’s risk of bias tool for animal studies [18], an adaptation of the Cochrane Risk of Bias tool specifically designed for animal intervention studies. Domains assessed included: selection bias (sequence generation, baseline characteristics, allocation concealment); performance bias (random housing, blinding of caregivers/investigators); detection bias (blinding of outcome assessors); and attrition bias (incomplete outcome data). Each domain was judged as low risk, high risk, or unclear risk by two independent reviewers, with disagreements resolved by consensus.

### Regulatory approval classification

For therapies that progressed to clinical trials, a strict classification system was applied to assess true translational success. Classifications were applied blinded to post-market safety outcomes by two reviewers (TRE, JEF) with a third reviewer (JPS) adjudicating disagreements. The classification categories were:

TRUE SUCCESS: Novel drug approved for the same indication tested in preclinical studies via modern regulatory pathway (post-1990 approval). This represents genuine predictive success of the animal model.

SUCCESS WITH CAVEATS: Approved for the same indication but with methodological limitations that complicate interpretation: predates modern trial requirements (grandfathered), represents a class effect rather than first-in-class (where earlier drugs in the class established the pathway), or cell therapy with different regulatory framework.

APPROVED–DIFFERENT INDICATION: Drug achieved regulatory approval but NOT for the indication tested in the preclinical studies. These approvals do not validate the animal model for the tested condition. For example, a drug tested preclinically for traumatic brain injury but approved only for transplant rejection does not demonstrate that the TBI animal model predicted human efficacy.

FAILED: Definitively failed in clinical trials (negative Phase II or Phase III results, development discontinued for efficacy reasons). This represents clear predictive failure of the animal model.

NOT APPROVED: Still experimental, insufficient evidence for regulatory submission, or nutraceutical/supplement without regulatory pathway.

REGIONAL ONLY: Limited approval in specific jurisdictions (e.g., Japan, South Korea) without US FDA or European Medicines Agency approval.

Devices and physical interventions (n = 17: 10 devices, 7 non-drug interventions including hypothermia and hyperbaric oxygen protocols) were classified separately and excluded from drug translation rate calculations to maintain a homogeneous denominator.

### Post-market safety assessment

For therapies classified as TRUE SUCCESS or SUCCESS WITH CAVEATS, comprehensive post-market safety surveillance was conducted to determine whether preclinical efficacy and safety predictions were replicated in real-world human use. This analysis was not specified in the original protocol (see S1 File, Section S2, Protocol Deviation #5) but was added during the regulatory classification phase when it became apparent that approval status alone was insufficient to characterise translational outcomes. The post-market safety criteria were predefined before any safety data were extracted. This analysis should therefore be considered exploratory and hypothesis-generating. We considered post-market safety concerns ‘major’ if they met at least one of the following five predefined criteria: (a) FDA black box warning or Public Health Notification issued after initial approval; (b) Clinical trial halted early by Data Safety Monitoring Board due to harm signal; (c) Significant mortality or serious morbidity signal identified in large randomised controlled trials or meta-analyses (relative risk >1.5); (d) Product liability litigation involving more than 1,000 patients or settlements exceeding $50 million; (e) Regulatory restriction, market withdrawal, or new contraindication added post-approval.

Data sources for post-market safety assessment included: FDA Adverse Event Reporting System (FAERS); FDA Drug Safety Communications and Public Health Notifications; published systematic reviews and meta-analyses of clinical trial safety data; US Senate and Congressional investigation reports (where applicable) [22]; PubMed literature searches for post-market safety studies; and legal databases for product liability litigation outcomes. The post-market evaluation was qualitative rather than meta-analytic; we did not claim comprehensive ascertainment of all adverse events but rather documented major safety signals meeting our predefined criteria.

### Data synthesis

The primary outcome was the proportion of preclinical studies demonstrating evidence of translation to human clinical trials. We present translation rates using multiple denominators and definitions to allow readers to assess how conclusions depend on definitional choices. Confidence intervals (95%) were calculated using the Wilson score method, which provides more accurate coverage for proportions near 0 or 1. Pre-specified subgroup analyses examined translation rates by therapy type, species (rat vs mouse vs other), injury model, and publication year (1990–1999 vs 2000–2010).

## Results

### Study selection

Database searches identified 3,847 records. After duplicate removal (n = 1,396), 2,451 records were screened by title and abstract, of which 1,028 were excluded as ineligible. Of 1,423 records proceeding to full-text review, 1,257 studies met inclusion criteria and were included in the final analysis. The 166 studies excluded at full-text review were removed for the following reasons: fundamental biology without therapeutic intervention (65%); not meeting population, intervention, or outcome criteria (24%); existing clinical therapy at time of publication (8%); and other reasons including predatory journals and non-English full text (3%). A complete list of excluded studies with reasons is provided in S1 File, Section S5. The PRISMA flow diagram is presented in Fig 1.

**Fig 1:**
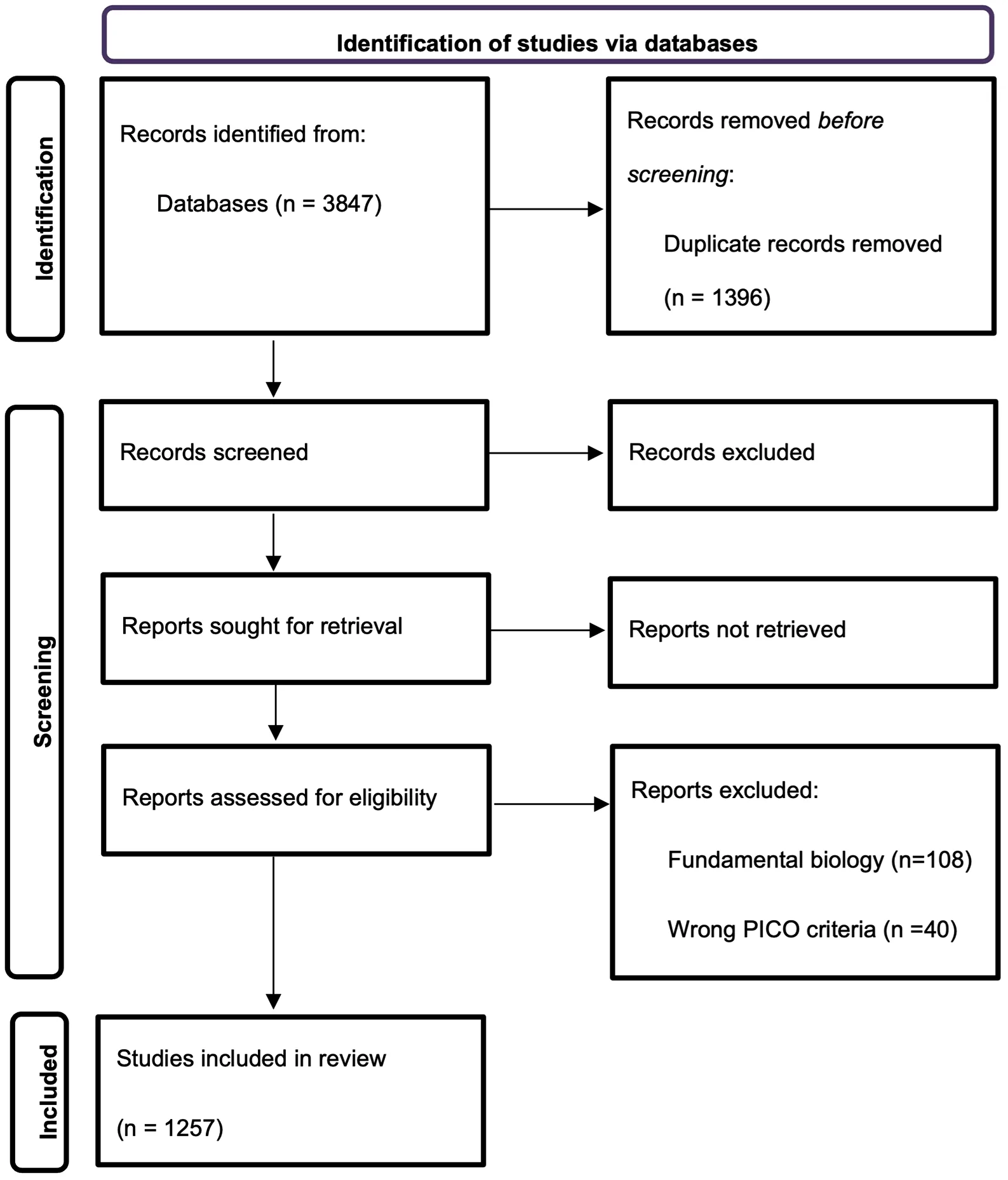
PRISMA 2020 flow diagram showing identification, screening, and inclusion of studies.

### Study characteristics

The 1,257 included studies predominantly used rat models (n = 882, 70.2%), followed by mice (n = 284, 22.6%), guinea pigs (n = 32, 2.5%), and other species including rabbits, hamsters, and multi-species studies (n = 59, 4.7%). Publication rates increased steadily over time, with peak publication years between 2007 and 2010 reflecting the growth of preclinical acute injury research during this period. The most common injury models studied were spinal cord injury (21.1%), lung injury/ARDS (20.9%), traumatic brain injury (11.0%), and radiation injury (8.9%). Full study characteristics are presented in S1 File, Table S1.

### Risk of bias in included studies

Risk of bias assessment revealed substantial methodological concerns across included studies (Table 1). Sequence generation (randomisation method) was adequate (low risk) in only 18% of studies, with 72% unclear and 10% high risk. Allocation concealment was rarely reported (low risk 8%, unclear 89%, high risk 3%). Blinding of outcome assessors was low risk in 34%, unclear in 58%, and high risk in 8%.

**Table 1:** Risk of bias assessment using SYRCLE domains (n = 1,257 studies).

Domain	Low Risk	Unclear	High Risk	n studies
1. Sequence generation	18%	72%	10%	1,257
2. Baseline characteristics	45%	48%	7%	1,257
3. Allocation concealment	8%	89%	3%	1,257
4. Random housing	12%	85%	3%	1,257
5. Blinding (investigators)	15%	78%	7%	1,257
6. Blinding (assessors)	34%	58%	8%	1,257
7. Incomplete outcome data	67%	28%	5%	1,257

Each domain was judged as low risk, high risk, or unclear risk by two independent reviewers. Domains assessed: sequence generation, baseline characteristics, allocation concealment, random housing, blinding of caregivers/investigators, blinding of outcome assessors, and incomplete outcome data.

### Headline translation rate

Of 1,257 included studies, 251 (20.0%; 95% CI 17.9–22.3%) demonstrated evidence of progression to human clinical trials for the same or related therapeutic indication.

### Regulatory approval analysis

From the 251 studies with evidence of translation to clinical trials, we identified 100 distinct therapies with sufficient clinical outcome data to classify regulatory status. Of these, 17 were devices or physical interventions (10 devices, 7 non-drug interventions including hypothermia and hyperbaric oxygen) which were analysed separately. Among the remaining 83 drug therapies, regulatory outcomes were distributed as shown in Table 2. Complete regulatory classifications for all 100 therapies are provided in S1 File, Table S2.

**Table 2:** Regulatory outcomes for 83 therapies with preclinical-to-clinical translation data.

Category	n	Rate	95% CI	Examples
TRUE SUCCESS	3	3.6%	1.2–10.1%	Palifermin, rhBMP-2, rhGH
SUCCESS WITH CAVEATS	4	4.8%	1.9–11.7%	Mannitol, pantoprazole, beractant, Epicel
APPROVED – DIFFERENT INDICATION	29	35%	25.6–45.7%	Minocycline, cyclosporin A, statins
FAILED	15	18%	11.3–27.7%	Progesterone, EPO, tirilazad, magnesium
NOT APPROVED	27	32.6%	23.4–43.2%	MSCs, resveratrol, curcumin
REGIONAL ONLY	5	6.0%	2.6–13.3%	Sivelestat, tanezumab
**Total**	**83**	**100%**		

Categories: TRUE SUCCESS (novel drug approved for the tested indication); SUCCESS WITH CAVEATS (approved for tested indication with methodological limitations); APPROVED–DIFFERENT INDICATION (approved for a different indication from that tested preclinically); FAILED (definitively failed in Phase II or Phase III clinical trials); NOT APPROVED (still experimental or no regulatory pathway); REGIONAL ONLY (limited jurisdictional approval without FDA or EMA approval). 95% confidence intervals calculated using Wilson score method. Categories are mutually exclusive.

The regulatory success rate for novel drug therapies, those achieving first-in-class approval for the indication actually tested in preclinical studies, was 3.6% (3/83; 95% CI 1.2–10.1%). When the 17 devices and physical interventions were included in the denominator (none achieved TRUE SUCCESS for tested indications), the rate was 3.0% (3/100). The rate rose to 8.4% (7/83) when drugs with methodological caveats (class effects, grandfathered approvals, cell therapies) were included in the success category.

Twenty-nine drugs (34.9%) were approved for indications entirely different from those tested in the preclinical studies. These approvals do not validate the predictive capacity of the animal models used for the original therapeutic targets. The therapies approved for different indications are detailed in S1 File, Table S5.

### Device and physical intervention outcomes

Among the 17 devices and physical interventions, none achieved TRUE SUCCESS for the indications tested in our preclinical dataset. Hypothermia protocols reported the highest translation-to-trial rate (69%) but remain experimental or guideline-inconclusive for most acute injury applications within our scope. Hyperbaric oxygen similarly progressed to clinical trials but lacks definitive regulatory approval for acute injury indications.

### Clinical trial failures

Fifteen therapies (18.1% of those progressing to trials) with robust preclinical evidence definitively failed in Phase II or Phase III clinical trials (Table 3). The four therapies with the highest preclinical-to-trial translation rates all failed or showed inconclusive results in definitive human trials [23–26]. Full details of all 15 clinical trial failures with trial citations are provided in S1 File, Table S3.

**Table 3:** Therapies with highest preclinical translation rates (≥50%) and their clinical outcomes.

Therapy	Preclinical indication	Preclinical studies (n)	Translation rate	Key trial(s)	Clinical outcome
Minocycline	SCI/TBI	7	71% (5/7)	Multiple Phase II	Inconclusive/negative
Hypothermia	TBI/perinatal asphyxia	16	69% (11/16)	Multiple RCTs	Inconclusive for most acute injury indications
Erythropoietin	TBI/SCI	17	59% (10/17)	EPO-TBI	No benefit; trend to harm
Progesterone	TBI	12	50% (6/12)	ProTECT III, SyNAPSe	No benefit vs placebo

Translation rate = proportion of included preclinical studies for each therapy that progressed to human clinical trial. Preclinical study counts derived from therapy grouping of 1,257 included studies. Clinical outcome classifications based on published Phase II or Phase III trial results.

### Post-market safety outcomes

In the exploratory post-market safety analysis, of the seven therapies classified as translational successes for the tested indication (TRUE SUCCESS n = 3; SUCCESS WITH CAVEATS n = 4), six (86%) met at least one of our predefined criteria for major post-market safety concerns (Table 4). Only beractant (Survanta®) demonstrated a relatively clean post-market safety profile. Detailed post-market safety data sources and findings are presented in S1 File, Table S4.

**Table 4:** Post-market safety outcomes for seven therapies classified as translational successes.

Therapy	(a)	(b)	(c)	(d)	(e)	Total	Key safety findings
***TRUE SUCCESS***							
Palifermin	✓	✓	–	–	✓	**3/5**	Trial stopped (worsened mucositis); narrowed to autologous only
rhBMP-2	✓	–	✓	✓	✓	**4/5**	AE rates 10–50 × higher; cancer RR 3.45; > $100M settlements
rhGH (burns)	–	✓	✓	–	✓	**3/5**	Mortality 44% vs 18% (RR 2.4); contraindicated in critically ill
***SUCCESS WITH CAVEATS***							
Mannitol	–	–	✓	–	✓	**2/5**	Renal failure risk; paradoxical oedema; NCS ‘unable to recommend’
Pantoprazole	✓	–	✓	✓	✓	**4/5**	5 FDA warnings; CKD + 20–50%; fractures +30%; $590M settlements
Beractant	–	–	–	–	–	**0/5**	Sepsis 20.7% vs 16.1%; relatively clean profile
Epicel*	–	–	–	–	✓	**1/5**	FDA: ‘effectiveness not demonstrated’; SCC warning added 2022

Criteria: (a) FDA black box warning or Public Health Notification; (b) Clinical trial stopped early for safety; (c) Relative risk >1.5 for serious adverse event in large RCT or meta-analysis; (d) Product liability litigation > $50 million; (e) Regulatory restriction or new contraindication post-approval. *Epicel: FDA explicitly states ‘effectiveness not demonstrated’; squamous cell carcinoma warning added 2022.

### Summary translation rates

Translation rates under all definitions are summarised in Table 5.

**Table 5:** Summary translation rates by multiple definitions.

Definition	n/N	Rate	95% CI	Denominator
Any trial entry (headline)	251/1,257	20.0%	17.9–22.3%	All studies
TRUE SUCCESS only	3/83	3.6%	1.2–10.1%	Drug therapies
+ SUCCESS WITH CAVEATS	7/83	8.4%	4.1–16.4%	Drug therapies
+ REGIONAL ONLY	12/83	14.5%	8.5–23.6%	Drug therapies
+ DIFFERENT INDICATION	41/83	49.4%	38.9–59.9%	Drug therapies
Including devices	3/100	3.0%	1.0–8.5%	All therapies
Novel therapies only	3/83	3.6%	1.2–10.1%	Drug therapies
Adjusted for post-market failures	1–2/83	1.2–2.4%	0.2–8.4%	Drug therapies

Definitions are cumulative; each row includes all categories above it. 95% confidence intervals calculated using Wilson score method.

## Discussion

Within the acute injury domain we examined, the commonly cited ‘20% translation rate’ requires substantial qualification. When stringent criteria are applied, requiring novel first-in-class regulatory approval for the indication actually tested in animal models, the true translation rate falls to 3.6% (95% CI 1.2–10.1%). Even among the seven drugs meeting any definition of translational success for the tested indication, six (86%) subsequently demonstrated major post-market safety concerns that preclinical studies failed to predict. Depending on definitional stringency, translation rates range from 1.2% (adjusted for post-market failures) to 20.0% (headline rate); we present multiple definitions to allow readers to assess which is most appropriate for their purposes.

### Why the headline rate requires qualification

The headline 20% translation rate conflates several distinct phenomena that have very different implications for assessing animal model validity. First, translation to trial initiation is treated as equivalent to translation to regulatory approval, when in fact most drugs that enter trials subsequently fail. Second, the headline rate includes repurposed drugs where the preclinical model is not truly being tested, the drug already had established human safety data from its original indication, so the animal model was not predicting novel human outcomes. Third, it does not distinguish between drugs approved for the indication tested preclinically versus those approved for entirely different conditions; the latter do not validate the animal model’s predictive capacity for the original therapeutic target. Fourth, it ignores post-market failures that reveal the preclinical predictions were incorrect.

### The high-translation paradox

Our subgroup analysis revealed a counterintuitive and important pattern: therapies with the highest preclinical-to-trial translation rates (minocycline 71%, hypothermia 69%, erythropoietin 59%, progesterone 50%) all subsequently failed or showed inconclusive results in definitive Phase II/III trials [23–26]. This finding aligns with systematic evidence that publication bias substantially inflates preclinical efficacy estimates [8,27,28]. When many laboratories publish positive results for a therapy, this may reflect selective reporting of model-specific effects rather than robust therapeutic potential that will generalise to human patients. The very success of these therapies in generating preclinical publications may have been a warning sign rather than an indicator of translational promise.

### Post-market safety failures and predictive validity

The post-market safety outcomes observed among the seven therapies classified as translational successes illustrate the limitations of regulatory approval as an endpoint for assessing preclinical predictive validity. Six of these seven therapies demonstrated major safety concerns that were not predicted by the preclinical studies supporting their development.

The case of rhBMP-2 (Infuse Bone Graft) is particularly instructive. Thirteen industry-sponsored trials involving 780 patients reported zero adverse events [29]. Independent systematic review subsequently identified adverse event rates substantially higher than industry reports had indicated [29–31], and the FDA issued a Public Health Notification in 2008 citing life-threatening complications including airway compression in off-label cervical spine use [32]. A cancer risk ratio of 3.45 (95% CI 1.98–6.00) at 24 months was identified in individual participant data meta-analysis, though this finding remains contested by subsequent large cohort studies [30,31]. Approximately 6,000 lawsuits were filed, resulting in settlements exceeding $300 million. The US Senate Finance Committee investigation documented total physician payments exceeding $210 million over 15 years from the manufacturer [22]. This case demonstrates not only a failure of preclinical models to predict human safety outcomes, but also how financial conflicts of interest can distort the evidence base upon which translation decisions are made.

Recombinant human growth hormone, tested preclinically for burns, demonstrated a 2.4-fold increased mortality in critically ill adults (44% vs 18%, p < 0.001) in a randomised trial of 532 ICU patients published in 1999 [33].28 The trial was terminated early by the Data Safety Monitoring Board. rhGH is now effectively contraindicated in critically ill adults, though paediatric use continues under careful monitoring [34].

Pantoprazole has been the subject of five distinct FDA safety communications since approval in 2000, none of which were predicted by preclinical studies. These include warnings for acute interstitial nephritis and chronic kidney disease (20–50% increased risk) [35,36], bone fractures, Clostridium difficile infection, and hypomagnesaemia. Product liability litigation has involved more than 18,600 plaintiffs with settlements totalling $590.4 million. Post hoc analysis of the COMPASS trial (n = 17,598) demonstrated significantly faster eGFR decline with pantoprazole compared to placebo [37].

Palifermin generated secondary malignancy concerns in the haematological malignancy population for which the drug was indicated, leading to enhanced monitoring requirements and restricted use recommendations. Mannitol demonstrated significant nephrotoxicity (acute kidney injury in 6–12% of treated patients) and paradoxical rebound cerebral oedema with prolonged administration, and a Cochrane review found that mannitol may increase the likelihood of death compared with hypertonic saline [38–40]. These complications have prompted clinical practice guidelines recommending hypertonic saline as an alternative in many settings [41].

Only beractant (Survanta®) demonstrated a relatively clean post-market safety profile, with post-treatment nosocomial sepsis (20.7% vs 16.1%, p = 0.019) not associated with increased mortality and no significant allergic reactions despite the bovine origin of the product.

When current clinical status is considered, the translational picture becomes starker still. Of the three TRUE SUCCESS therapies, rhGH is effectively contraindicated for the tested indication (critical illness), rhBMP-2 remains technically available for its approved indication (anterior lumbar fusion with LT-CAGE) but clinical use has declined dramatically following the FDA Public Health Notification, litigation, and exposure of systematic adverse event underreporting, and palifermin carries enhanced monitoring requirements and restricted use recommendations due to secondary malignancy concerns in the population for which it was indicated. Among the SUCCESS WITH CAVEATS therapies, mannitol has been supplanted by hypertonic saline in clinical guidelines for many acute injury indications, pantoprazole carries five FDA safety communications, and Epicel® remains available only under humanitarian device exemption with the FDA explicitly stating that effectiveness has not been demonstrated. Only beractant remains in routine clinical use for the tested indication without major safety caveats, and as a class-effect surfactant replacement rather than a novel first-in-class translation, it does not represent a genuinely novel predictive success of the preclinical model. In summary, of 83 drug therapies tested in small rodent acute injury models, not one achieved novel first-in-class regulatory approval and remains in routine clinical use for the tested indication with a favourable safety profile.

These findings illuminate a fundamental limitation of rodent models: their systematic failure to predict human safety outcomes. The preclinical studies that supported development of rhBMP-2, rhGH, and other therapies showed efficacy without signalling the serious adverse events that emerged in human populations. This predictive failure extends beyond efficacy translation to encompass safety translation, a dimension rarely examined in conventional translational assessments but arguably more consequential for patient welfare.

### Alternative explanations and limitations

We acknowledge that poor translation rates may reflect problems beyond animal model validity per se. Clinical trial design issues, including inappropriate patient selection, suboptimal dosing regimens, and insufficient statistical power, may contribute to translational failure even when the underlying biology is sound. Other factors, however, are better understood as reflections of the failure of animal models to translate rather than as influences separate from it: differences in drug pharmacokinetics between rodents and humans, the timing of intervention relative to injury, the inherent challenges of matching preclinical models to heterogeneous human conditions, and publication bias in the preclinical literature that inflates apparent efficacy and sets unrealistic expectations for clinical trials.

Our data cannot fully separate these contributing factors. Nonetheless, the finding that preclinical evidence was consistently over-optimistic relative to clinical outcomes, regardless of whether the limiting factor lay in model selection, trial design, or implementation, indicates that the current pathway from preclinical promise to human benefit has substantial limitations in this therapeutic area.

### Methodological limitations

This study has several important methodological limitations that should inform interpretation of our findings.

Database coverage: We searched only PubMed/MEDLINE. Studies indexed exclusively in EMBASE, Web of Science, or other databases may have been missed. However, sensitivity checks for three exemplar therapies identified no additional eligible studies, and the high overlap between PubMed and other databases for preclinical rodent literature suggests our estimates are likely conservative rather than inflated [20].

### Contemporary relevance of the findings

Historical timeframe and contemporary relevance: The 1990–2010 publication window was necessary to allow sufficient follow-up time for translation assessment. However, contrary to expectations that contemporary practices might differ substantially, multiple lines of evidence suggest our findings remain directly relevant to current preclinical research.

First, the same rodent acute injury models examined in our review remain in widespread use. A 2024 systematic review identified 4,948 articles on rodent traumatic brain injury models published between 2014 and 2024, employing the same controlled cortical impact, fluid percussion, and weight drop models used throughout our study period [42]. Similarly, over 80% of preclinical spinal cord injury studies continue to use thoracic contusion models despite cervical injuries accounting for approximately 50% of clinical cases, a mismatch that persists from our study era [43].

Second, despite the introduction of reporting guidelines including ARRIVE (2010, updated 2020) [44] and NIH principles for preclinical research (2014), methodological rigour has not substantively improved [45,46]. Macleod et al. surveyed in vivo research from leading UK institutions and found very limited reporting of measures to reduce risk of bias, with significantly lower reporting of randomisation in high-impact journals [47]. A nationwide Danish investigation comparing preclinical studies from 2009 and 2018 found only modest improvements: reporting of randomisation increased from 24% to 41%, blinded outcome assessment from 24% to 38%, but blinded experiment conduct remained at 2–4%, and the method of random allocation was reported in just 1–6% of studies [48]. Most strikingly, Townsend et al. analysed a stratified random sample of comparative laboratory animal experiments published in 2022 across North America and Europe and found that as few as 0–2.5% utilised valid, unbiased experimental designs [49]. A study titled “ARRIVE has not ARRIVEd” found that journal endorsement of these guidelines did not improve reporting quality in animal research [50,51]. The ARRIVE 2.0 update itself acknowledged that “*adherence to the guidelines has been inconsistent, and the anticipated improvements in the quality of reporting in animal research publications have not been achieved*.” [44]. The methodological weaknesses we identified (adequate randomisation in only 18% of studies, allocation concealment unclear in 89%) are therefore not historical artefacts but reflect ongoing systemic deficiencies in preclinical experimental design.

Third, and most significantly, translation failure rates have not improved and may have worsened. Over 90% of investigational drugs continue to fail during clinical development [52], with one analysis noting that “*despite efforts to improve the predictability of animal testing, the failure rate has actually increased*.” [52,53]. In traumatic brain injury specifically, a 100% failure rate for neuroprotective agents persists: progesterone, which showed robust efficacy across dozens of rodent studies, failed in two large Phase III trials (ProTECT III, n = 882; SyNAPSe, n = 1,195) in 2014 [23,24]. Erythropoietin, minocycline, and therapeutic hypothermia, the highest-translating therapies in our dataset, have similarly failed or shown inconclusive results in definitive human trials. No rodent acute injury model has been abandoned or substantially modified as a consequence of these failures.

Fourth, the “*valley of death*” between preclinical and clinical research has been described as “*widening and getting deeper*” rather than narrowing [53]. The likelihood of FDA approval for drugs entering clinical trials remains approximately 10–14%, unchanged from historical benchmarks [10]. The FDA Modernization Act 2.0 (2022), which removed the mandatory requirement for animal testing before human trials, reflects regulatory acknowledgment that preclinical animal studies have not achieved their intended predictive function [54].

Our findings should therefore be interpreted not as historical artefact but as documentation of fundamental limitations in small rodent acute injury models that persist to the present day. The continued use of these models without substantial modification, despite decades of translational failure, raises important questions about the scientific basis for their ongoing regulatory acceptance.

### Rationale for post-market safety assessment

Post-market safety assessment methodology and rationale: Our post-market safety evaluation employed qualitative synthesis rather than meta-analysis, and we do not claim comprehensive ascertainment of all adverse events. However, this assessment represents a critical and novel component of translational evaluation that is systematically absent from conventional success rate calculations.

The 1990–2010 publication window was deliberately selected to permit adequate post-market follow-up. Evidence demonstrates that serious adverse drug reactions frequently emerge only after extended market exposure: a landmark JAMA study found that half of all new black box warnings occurred within 7 years of drug introduction, while the estimated probability of acquiring a new black box warning or market withdrawal reached 20% over 25 years of follow-up [15]. More recent analyses confirm that approximately one-third of novel therapeutics approved between 2001 and 2010 required significant post-marketing safety actions, most commonly the addition of new black box warnings [16]. Drugs approved through accelerated pathways are 3.5 times more likely to receive post-market black box warnings [55]. By examining therapies with 15–35 years of post-market exposure, our analysis captures safety signals that would be invisible in shorter follow-up periods.

This systematic pattern of post-market safety failures has profound implications for interpreting apparent preclinical success. Traditional translation rate calculations count a therapy as ‘successful’ at the point of regulatory approval, yet our analysis demonstrates that this endpoint fails to capture the ultimate clinical utility of the therapy. When post-market safety failures are incorporated, the proportion of rodent acute injury research that generated clinically beneficial human therapies approaches 1.2% (1 of 83 drug therapies) rather than the 3.6% calculated from regulatory approval alone.

### Additional limitations

Scope: We focused exclusively on acute injury in small rodent models. Translation rates may differ substantially for other therapeutic areas (oncology, infectious disease), other species (large animals, non-human primates), or other injury types (chronic conditions). Extrapolation beyond the domain we examined should be cautious.

Classification subjectivity: Our regulatory classification criteria, while predefined and applied by independent reviewers, inevitably involve judgment. We have presented multiple definitions and translation rates to allow readers to assess how conclusions depend on definitional choices. Sensitivity analyses examining database coverage and classification robustness are presented in S1 File, Section S6.

Registration: This review was registered on the Open Science Framework (https://osf.io/w97fd). Prospective PROSPERO registration was not possible as animal systematic reviews were not routinely accepted at the time of protocol development.

### Implications

These findings raise important questions about the current translational research paradigm in acute injury, though we are cautious about drawing strong policy conclusions from a single systematic review focused on one therapeutic domain. The discrepancy between headline translation rates (20%) and true regulatory success rates (3.6%) suggests that commonly cited figures may give a misleading impression of preclinical model predictive validity. The observation that therapies with the highest preclinical translation rates all failed clinically suggests that robust preclinical evidence alone may not predict human outcomes.

Whether animal research in this area should continue is a question our data can inform but not settle. What follows from a 3.6% regulatory translation rate is that the burden of justification now rests with those proposing further small rodent studies of acute injury therapies, and that the harm imposed should be weighed against that rate when designing translational research programmes and when communicating expectations to patients, funders, and policymakers. Constructive responses might include: greater emphasis on understanding why specific models fail to translate; complementary use of human tissue models, organoids, and computational approaches; more rigorous preclinical methodology including pre-registration, blinding, and multi-centre replication; and carefully designed early-phase human studies to test translational hypotheses before large resource commitments.

## Conclusions

Within the acute injury domain examined, the true regulatory success rate for genuinely novel preclinical therapies is 3.6%, not 20%. Depending on definitional stringency, rates range from 1.2% to 20.0%; we present multiple definitions to allow readers to assess which is most appropriate for their purposes.

Among therapies achieving any form of approval for the tested indication, the majority (6/7, 86%) demonstrated major post-market safety concerns including mortality signals, adverse event rates substantially higher than industry reports indicated, and hundreds of millions of dollars in litigation settlements.

In all small rodent acute injury studies from 1990 to 2010, we did not identify any drug that both translated as a novel first-in-class therapy and maintained a favourable post-market safety profile without major caveats, though beractant came closest with a relatively clean safety record.

These findings indicate that, within acute injury research, small rodent models have limited demonstrated predictive validity for human therapeutic outcomes. Critical re-evaluation of current translational research paradigms in this field appears warranted.

## Supporting information

S1 File Supporting information. Section S1, PRISMA 2020 checklist for systematic reviews; Section S2, protocol deviations log; Section S3, complete search strategy documentation; Section S4, AI-assisted validation protocol and results; Section S5, excluded studies with reasons (n = 1,194); Section S6, sensitivity analyses results; Table S1, characteristics of the 1,257 included studies; Table S2, complete therapy classification database (100 therapies); Table S3, fifteen clinical trial failures with trial citations; Table S4, post-market safety data sources and detailed findings; Table S5, twenty-nine drugs approved for indications other than the one tested preclinically.
(DOCX)

S2 File PRISMA 2020 abstract checklist. (DOCX)
