# Supplementary material for: From preclinical promise to regulatory reality: translation of small rodent acute injury therapies to human approval and safety outcomes: A systematic review
Source: PLoS One. 2026 Sep 28;21(9):e0358773. doi: 10.1371/journal.pone.0358773 (PMC13618947; doi:10.1371/journal.pone.0358773)
Supplement: Supplementary file 1 [file pone.0358773.s001.docx]

**S1 File. Supporting information.**

**Section S1** - **PRISMA 2020 Checklist for Systematic Reviews**

From: Page MJ, McKenzie JE, Bossuyt PM, et al. The PRISMA 2020 statement: an updated guideline for reporting systematic reviews. BMJ 2021;372:n71. doi: 10.1136/bmj.n71

| **Section** | **Item** | **Checklist item** | **Location** |
| --- | --- | --- | --- |
| **TITLE** | 1 | Identify the report as a systematic review | Title, p.1 |
| **ABSTRACT** | 2 | See the PRISMA 2020 for Abstracts checklist | p.2 |
| **INTRODUCTION** | 3 | Rationale: Describe the rationale for the review in the context of existing knowledge | pp.3-4 |
|  | 4 | Objectives: Provide an explicit statement of the objective(s) or question(s) the review addresses | p.4 |
| **METHODS** | 5 | Eligibility criteria: Specify the inclusion and exclusion criteria for the review | pp.4-5 |
|  | 6 | Information sources: Specify all databases, registers, websites, organisations, reference lists and other sources searched | p.5; S3 |
|  | 7 | Search strategy: Present the full search strategies for all databases | S3 |
|  | 8 | Selection process: Specify methods for deciding study eligibility | pp.5-6 |
|  | 9 | Data collection process: Specify methods for data extraction | p.6 |
|  | 10a | Data items: List and define all outcomes for which data were sought | pp.6-7 |
|  | 10b | Data items: List and define all other variables for which data were sought | p.7; Table S2 |
|  | 11 | Study risk of bias assessment: Specify methods for assessing risk of bias | p.6; Table 1 |
|  | 12 | Effect measures: Specify for each outcome the effect measure(s) used | p.8 |
|  | 13a-f | Synthesis methods: Describe processes for deciding eligibility, preparing data, pooling, heterogeneity, sensitivity analyses | p.8 |
|  | 14 | Reporting bias assessment: Describe methods for assessing publication bias | p.8; S6 |
|  | 15 | Certainty assessment: Describe methods for assessing certainty of evidence | N/A* |

| **RESULTS** | 16a | Study selection: Describe results of search and selection process | p.9; Fig 1 |
| --- | --- | --- | --- |
|  | 16b | Study selection: Cite studies excluded at full-text with reasons | p.9; S5 |
|  | 17 | Study characteristics: Cite each included study and present its characteristics | p.9; Table S1 |
|  | 18 | Risk of bias in studies: Present assessments of risk of bias | p.9; Table 1 |
|  | 19 | Results of individual studies: Present all results for all outcomes | pp.9-12; Tables 1-3 |
|  | 20a-d | Results of syntheses: Present results of syntheses including heterogeneity | pp.10-12; Table 5 |
|  | 21 | Reporting biases: Present assessments of risk of reporting biases | p.12 |
|  | 22 | Certainty of evidence: Present assessments of certainty for each outcome | N/A* |
| **DISCUSSION** | 23a | Discussion: Provide a general interpretation of results in context | pp.13-14 |
|  | 23b | Discussion: Discuss any limitations of evidence and review process | pp.14-15 |
|  | 23c | Discussion: Discuss implications of results for practice, policy, research | p.15 |
|  | 23d | Discussion: Discuss implications for future research | p.15 |
| **OTHER** | 24a | Registration and protocol: Provide registration information | p.4; S2 |
|  | 24b | Registration and protocol: Indicate where protocol can be accessed | p.4; OSF |
|  | 24c | Registration and protocol: Describe and explain protocol amendments | S2 |
|  | 25 | Support: Describe sources of financial or non-financial support | p.16 |
|  | 26 | Competing interests: Declare any competing interests of review authors | p.16 |
|  | 27 | Availability of data, code and other materials | p.16 |

**N/A: GRADE certainty assessment was not performed as this was a descriptive translation analysis rather than an intervention effectiveness review.*

*S2 = Section S2 (Protocol Deviations); S3 = Section S3 (Search Strategy); S5 = Section S5 (Excluded Studies); S6 = Section S6 (Sensitivity Analyses)*

# **Section S2 - Protocol Deviations Log**

This document records all deviations from the original protocol (dated March 2022) with rationale and assessment of potential impact on review findings.

| **#** | **Deviation** | **Description** | **Rationale** | **Impact Assessment** |
| --- | --- | --- | --- | --- |
| 1 | Database restriction | Protocol specified "at least two databases"; review used PubMed only | Resource constraints; PubMed provides comprehensive coverage for preclinical rodent literature | Low - sensitivity check of Embase for 3 exemplar therapies identified no additional eligible studies |
| 2 | Language restriction | Protocol did not specify language limits; English-only applied | Resource constraints for translation; majority of indexed preclinical literature is English | Low - 49 non-English articles excluded represents 4.1% of exclusions |
| 3 | PROSPERO registration | Protocol recommended prospective registration; not registered | PROSPERO did not routinely accept animal SR protocols at time of protocol development | Moderate - addressed by documenting protocol a priori and reporting deviations transparently |
| 4 | Risk of bias tool | Protocol specified SYRCLE tool; applied with modifications | Some domains not applicable to all study types in dataset | Low - modifications documented; core bias domains assessed |
| 5 | Post-market assessment | Not specified in original protocol; added during analysis | Emerged as critical finding during regulatory classification | Moderate - post-hoc addition; however, methods predefined before safety data extracted |
| 6 | AI validation | Not specified in original protocol; added for methodological rigour | Opportunity to strengthen screening validation using emerging methods | Low - supplementary validation; does not affect primary findings |
| 7 | Therapy grouping | Protocol specified individual study analysis; therapies grouped | Multiple studies per therapy required aggregation for regulatory outcome mapping | Low - grouping criteria predefined and applied consistently |
| 8 | Date of search update | Original search March 2022; no update search conducted | Historical cohort (1990-2010) would not be affected by update search | None - date restriction means no new eligible studies possible |

**Section S3** - **Complete Search Strategy Documentation**

**1. Database Information**

| **Database** | MEDLINE via PubMed |
| --- | --- |
| **Platform** | National Library of Medicine (https://pubmed.ncbi.nlm.nih.gov) |
| **Date of Search** | 22 March 2022 |
| **Date Range** | 1 January 1990 to 31 December 2010 |
| **Language Limits** | English only |

**2. Search Concept Framework**

The search strategy was constructed using five conceptual building blocks combined with Boolean operators:

| **Concept** | **Purpose** | **Key Terms** |
| --- | --- | --- |
| Acute | Identify acute (vs chronic) conditions | acute wound*, acute injur*, acute trauma* |
| Wound/Injury | Identify injury/trauma literature | wound*, injur*, trauma* |
| Therapy | Identify therapeutic interventions | therap*, heal*, care, treatment*, manag*, drug*, medication*, regimen* |
| Animal | Limit to animal model studies | animal*, rat, rats, mouse, mice, rodent*, guinea pig*, hamster*, rabbit* |
| Human | Sensitivity checking for translation | human trial*, human model*, human stud*, clinical trial*, clinical stud* |

**3. Building Block Definitions**

**Set 1: Acute Injury (Phrase Search)**

"acute wound*" OR "acute injur*" OR "acute trauma*"

**Set 2: Acute Injury (Adjacency Search)**

(acute AND (wound* OR injur* OR trauma*))

**Set 3: Therapy Terms**

therap* OR heal* OR care OR treatment* OR manag* OR drug* OR medication* OR regimen*

**Set 4: Animal Model Terms**

animal* OR rat OR rats OR rodent* OR mouse OR mice OR "guinea pig*" OR hamster* OR rabbit*

**Set 5: Human Translation Terms (Phrase)**

"human trial*" OR "human model*" OR "human stud*"

**Set 6: Human Translation Terms (Clinical)**

(human* AND ("clinical trial*" OR "clinical stud*" OR "clinical research"))

**Set 7: Human Translation Terms (General)**

(human AND (trial* OR model* OR stud*))

**4. Combined Search Strategies**

The following combinations were executed to maximise sensitivity:

**Set 8: Phrase Acute + Therapy + Animal**

((("acute wound*" OR "acute injur*" OR "acute trauma*")) AND ((therap* OR heal* OR care OR treatment* OR manag* OR drug* OR medication* OR regimen*))) AND ((animal* OR rat OR rats OR rodent* OR mouse OR mice OR "guinea pig*" OR hamster* OR rabbit*)) AND (("1990"[Date - Publication] : "2010"[Date - Publication]))

**Set 9: Adjacency Acute + Therapy + Animal**

(((acute AND (wound* OR injur* OR trauma*))) AND (therap* OR heal* OR care OR treatment* OR manag* OR drug* OR medication* OR regimen*)) AND (animal* OR rat OR rats OR rodent* OR mouse OR mice OR "guinea pig*" OR hamster* OR rabbit*) AND (("1990"[Date - Publication] : "2010"[Date - Publication]))

**Set 10: Phrase Acute + Therapy + Human Phrase**

((("acute wound*" OR "acute injur*" OR "acute trauma*") AND (therap* OR heal* OR care OR treatment* OR manag* OR drug* OR medication* OR regimen*)) AND ("human trial*" OR "human model*" OR "human stud*")) AND (("1990"[Date - Publication] : "2010"[Date - Publication]))

**Set 11: Adjacency Acute + Therapy + Human Phrase**

((((acute AND (wound* OR injur* OR trauma*))) AND (therap* OR heal* OR care OR treatment* OR manag* OR drug* OR medication* OR regimen*)) AND ("human trial*" OR "human model*" OR "human stud*")) AND (("1990"[Date - Publication] : "2010"[Date - Publication]))

**Set 12: Phrase Acute + Therapy + Human Clinical**

((("acute wound*" OR "acute injur*" OR "acute trauma*") AND (therap* OR heal* OR care OR treatment* OR manag* OR drug* OR medication* OR regimen*)) AND ((human* AND ("clinical trial*" OR "clinical stud*" OR "clinical research")))) AND (("1990"[Date - Publication] : "2010"[Date - Publication]))

**Set 13: Adjacency Acute + Therapy + Human Clinical**

(((acute AND (wound* OR injur* OR trauma*))) AND (therap* OR heal* OR care OR treatment* OR manag* OR drug* OR medication* OR regimen*)) AND ((human* AND ("clinical trial*" OR "clinical stud*" OR "clinical research"))) AND (("1990"[Date - Publication] : "2010"[Date - Publication]))

**Set 14: Phrase Acute + Therapy + Human General**

((("acute wound*" OR "acute injur*" OR "acute trauma*") AND (therap* OR heal* OR care OR treatment* OR manag* OR drug* OR medication* OR regimen*)) AND ((human AND (trial* OR model* OR stud*)))) AND (("1990"[Date - Publication] : "2010"[Date - Publication]))

**Set 15: Adjacency Acute + Therapy + Human General**

(((acute AND (wound* OR injur* OR trauma*))) AND (therap* OR heal* OR care OR treatment* OR manag* OR drug* OR medication* OR regimen*)) AND ((human AND (trial* OR model* OR stud*))) AND (("1990"[Date - Publication] : "2010"[Date - Publication]))

**5. Search Results Summary**

| **Search Set** | **Records** | **Focus** |
| --- | --- | --- |
| Sets 8-9 (Animal) | 2,847 | Primary preclinical literature |
| Sets 10-15 (Human) | 1,000 | Translation sensitivity check |
| **Total before deduplication** | **3,847** |  |
| Duplicates removed | 1,396 |  |
| **Records screened** | **2,451** |  |

**6. Hand Searching**

Reference lists of included studies and relevant systematic reviews were screened for additional eligible studies. No additional studies meeting inclusion criteria were identified through reference list screening.

**7. Sensitivity Analysis**

A targeted sensitivity check was conducted in Embase for three exemplar therapies (progesterone, erythropoietin, minocycline) to assess potential database coverage gaps. No additional eligible preclinical studies beyond those captured by our PubMed search were identified.

# **Section S4: AI Validation Protocol and Results**

## 1. Overview

AI-assisted validation was conducted to independently verify human screening decisions. A large language model (Claude, Anthropic) was used to classify a stratified random sample of studies using identical eligibility criteria provided to human reviewers.

## 2. Sampling Methodology

A stratified random sample of 200 studies was generated using a fixed random seed (seed = 42) to ensure reproducibility. The sample comprised 100 studies from the inclusion dataset (n = 1,257) and 100 studies from the exclusion dataset (n = 1,194). Sampling was performed using Python's pandas library with the rando]m_state parameter set to 42.

## 3. Classification Protocol

Each study was classified using the following eligibility criteria:

**Include if ALL criteria are met:**

- Small animal model (rat, mouse, guinea pig, hamster, rabbit) of acute injury
- Therapeutic intervention tested
- Efficacy outcomes with comparator
- Published 1990-2010
- Original research in English

**Exclude if ANY criteria apply:**

- Fundamental biology study without therapeutic intervention
- Does not meet population/intervention/outcome criteria
- Tests therapy already in routine clinical use at publication
- Review, editorial, commentary, or case report
- No comparator/control group
- In vitro study only (no in vivo animal component)

## 4. Results

AI validation of 200 randomly sampled studies demonstrated almost perfect agreement with human screening decisions.

| **Metric** | **Value** |
| --- | --- |
| Studies validated | 200 |
| Percentage agreement | 99.0% |
| Cohen's kappa (95% CI) | 0.98 (0.94–1.00) |
| Disagreements for re-review | 2 |

**Confusion Matrix:**

|  | **AI: Include** | **AI: Exclude** |
| --- | --- | --- |
| **Human: Include** | 99 | 1 |
| **Human: Exclude** | 1 | 99 |

## 5. Disagreement Analysis

Two studies were flagged by the AI classifier as potentially meeting exclusion criterion 3 (therapy already in clinical use at publication):

**Lidocaine study:** AI noted lidocaine was an established clinical anaesthetic. Re-review confirmed the study specifically investigated a novel neuroprotective application in traumatic brain injury, not the drug's established anaesthetic use.

**Indomethacin study:** AI noted indomethacin was an established NSAID. Re-review confirmed the study investigated a novel radioprotective application in acute radiation-induced gastrointestinal injury, distinct from the drug's approved indications.

Following re-review, both original human decisions were confirmed. The studies appropriately met inclusion criteria as they tested existing drugs for novel therapeutic applications in acute injury models not previously evaluated in clinical practice.

## 6. Interpretation

Kappa interpretation follows Landis and Koch (1977) guidelines:

< 0.00: Poor; 0.00–0.20: Slight; 0.21–0.40: Fair; 0.41–0.60: Moderate; 0.61–0.80: Substantial; 0.81–1.00: Almost perfect agreement.

The obtained kappa of 0.98 indicates almost perfect agreement between human and AI classifiers, supporting the reliability of the human screening decisions. The two disagreements were resolved by consensus re-review with 100% confirmation of original human decisions.

## 7. References

Landis JR, Koch GG. The measurement of observer agreement for categorical data. Biometrics. 1977;33(1):159-174.

# **Section S5 - Excluded Studies with Reasons (n=1,194)**

## Summary of Exclusion Reasons

| Exclusion Reason | n | % |
| --- | --- | --- |
| Fundamental biology study | 789 | 66.1 |
| Does not meet search criteria | 129 | 10.8 |
| Review/not original research | 106 | 8.9 |
| Existing clinical therapy at publication | 101 | 8.5 |
| Non-English article | 49 | 4.1 |
| Not a therapy R&D study | 13 | 1.1 |
| Human study (not animal) | 5 | 0.4 |
| Wrong species (not small rodent) | 2 | 0.2 |

## Complete List of Excluded Studies

Studies are listed alphabetically by first author surname (extracted from title where author field not available).

| **#** | **Title** | **Year** | **Exclusion Reason** |
| --- | --- | --- | --- |
| 1 | Abrogation of resistance to Theiler's virus-induced demyelination in C57BL mice by total body irradi... | 1990 | Fundamental biology study |
| 2 | Acute aggregation of serotonin-immunoreactive platelets in the injured spinal cord of rat and change... | 1990 | Fundamental biology study |
| 3 | Acute erosions of the gastric mucosa in burned rats: effect of gastric acidity and fluid replacement | 1990 | Fundamental biology study |
| 4 | Acute exposure to cigarette smoke induces airway hyperresponsiveness without airway inflammation in ... | 1990 | Fundamental biology study |
| 5 | Alternation of gastric mucosal glycoprotein (lectin-binding pattern) in gastric mucosa in stress. A ... | 1990 | Fundamental biology study |
| 6 | Anesthesia influences the outcome from experimental spinal cord injury | 1990 | Fundamental biology study |
| 7 | Argon laser irradiation of the otolithic organ | 1990 | Does not meet search criteria |
| 8 | Chronic cervical spinal cord injury and autonomic hyperreflexia in rats | 1990 | Fundamental biology study |
| 9 | Effects of phorbol myristate acetate-stimulated human leukocytes on rat lung | 1990 | Does not meet search criteria |
| 10 | Induction of diabetes in PVG/c strain rats by manipulation of the immune system | 1990 | Fundamental biology study |
| 11 | Interferons and collagen production | 1990 | Review/not original research |
| 12 | Intestinal complications following accelerated fractionated x-irradiation. An experimental study in ... | 1990 | Fundamental biology study |
| 13 | Mechanisms underlying the recovery of urinary bladder function following spinal cord injury | 1990 | Does not meet search criteria |
| 14 | Primary herpes simplex virus (type 1) infection delays healing of oral excisional and extraction wou... | 1990 | Fundamental biology study |
| 15 | Proliferation and differentiation of erythroleukemia cell line (ELM-I-1) in response to erythropoiet... | 1990 | Does not meet search criteria |
| 16 | Role of skin in the burn-induced reduction of reticuloendothelial phagocytic activity in rats | 1990 | Fundamental biology study |
| 17 | Traumatic tattoo removal: comparison of four treatment methods in an animal model with correlation t... | 1990 | Does not meet search criteria |
| 18 | Animal models for the study of fetal tissue repair | 1991 | Review/not original research |
| 19 | Cellular basis of ventricular remodeling after myocardial infarction | 1991 | Review/not original research |
| 20 | Cerebrovascular changes in a rat model of moderate closed-head injury | 1991 | Fundamental biology study |
| 21 | Damage and repair of mouse bronchial epithelium following acute inhalation of trichloroethylene | 1991 | Fundamental biology study |
| 22 | Degradation of and tissue reaction to biodegradable poly(L-lactide) for use as internal fixation of ... | 1991 | Existing clinical therapy at publication |
| 23 | Early and late effects of cisplatin and radiation at acute and low dose rates on the mouse skin and ... | 1991 | Existing clinical therapy at publication |
| 24 | Effects of hyperthermia applied to previously irradiated cervical spinal cord in the rat | 1991 | Review/not original research |
| 25 | Effects of pirenzepine on acute mucosal erosions, gastric acid and mucosal blood flow in the spinal ... | 1991 | Existing clinical therapy at publication |
| 26 | Epidermal growth factor in protection, repair, and healing of gastroduodenal mucosa | 1991 | Review/not original research |
| 27 | Evidence for a neural source of acute accumulation of serotonin in platelets in the injured spinal c... | 1991 | Fundamental biology study |
| 28 | Immune suppression after acute ethanol ingestion and thermal injury | 1991 | Fundamental biology study |
| 29 | Infusion of enteral vs parenteral nutrients using high-concentration branch-chain amino acids: effec... | 1991 | Existing clinical therapy at publication |
| 30 | Interactions in recovery and in residual injury from sequential treatments of mouse haemopoietic and... | 1991 | Existing clinical therapy at publication |
| 31 | Lung injury and complement activation: role of neutrophils and xanthine oxidase | 1991 | Does not meet search criteria |
| 32 | Murine radiation myeloid leukaemogenesis: a possible role for radiation-sensitive sites on chromosom... | 1991 | Fundamental biology study |
| 33 | Preclinical and clinical studies with recombinant human basic fibroblast growth factor | 1991 | Does not meet search criteria |
| 34 | Role of acid milieu in the gastroprotective and ulcer-healing activity of sucralfate | 1991 | Existing clinical therapy at publication |
| 35 | Role of vascular factors, including angiogenesis, in the mechanisms of action of sucralfate | 1991 | Existing clinical therapy at publication |
| 36 | Urinary excretion of iohexol after enteral administration in rats with radiation injury of the small... | 1991 | Existing clinical therapy at publication |
| 37 | Acute intraoperative arterial lengthening for closure of large vascular gaps | 1992 | Existing clinical therapy at publication |
| 38 | Acute ozone-induced change in airway permeability: role of infiltrating leukocytes | 1992 | Fundamental biology study |
| 39 | Adenine nucleotides stimulate migration in wounded cultures of kidney epithelial cells | 1992 | Does not meet search criteria |
| 40 | Alcohol exacerbates behavioral and neurochemical effects of rat spinal cord trauma | 1992 | Fundamental biology study |
| 41 | An animal model of chronic tympanic membrane perforation | 1992 | Fundamental biology study |
| 42 | Basic fibroblast growth factor mRNA increases in specific brain regions following convulsive seizure... | 1992 | Fundamental biology study |
| 43 | Biocompatibility studies on plasma polymerized interface materials encompassing both hydrophobic and... | 1992 | Fundamental biology study |
| 44 | Chronic pain-related syndrome in rats after ischemic spinal cord lesion: a possible animal model for... | 1992 | Fundamental biology study |
| 45 | Core body temperature responses immediately after cutaneous thermal injury in rats | 1992 | Fundamental biology study |
| 46 | Development of an orofacial model of acute inflammation in the rat | 1992 | Fundamental biology study |
| 47 | Elucidating the vascular response to burns with a new rat mode | 1992 | Fundamental biology study |
| 48 | Fetal cell grafts into resection and contusion/compression injuries of the rat and cat spinal cord | 1992 | Review/not original research |
| 49 | In vivo effect of insulin on the acute proliferative response of the rat aorta to injury | 1992 | Fundamental biology study |
| 50 | Local cerebral blood flow mapping before and after removal of acute subdural hematoma in the rat | 1992 | Fundamental biology study |
| 51 | Methylprednisolone does not decrease eicosanoid concentrations or edema in brain tissue or improve n... | 1992 | Existing clinical therapy at publication |
| 52 | Nerve tension and blood flow in a rat model of immediate and delayed repairs | 1992 | Fundamental biology study |
| 53 | Pseudomonas aeruginosa exotoxin A: its role in retardation of wound healing: the 1992 Lindberg Award | 1992 | Fundamental biology study |
| 54 | Pulmonary sequelae of intraparenchymal bismuth subgallate | 1992 | Existing clinical therapy at publication |
| 55 | Radioreductive effect of bestatin (Ubenimex) in BALB/c mice | 1992 | Existing clinical therapy at publication |
| 56 | Susceptibility of brain to AMPA induced excitotoxicity transiently peaks during early postnatal deve... | 1992 | Fundamental biology study |
| 57 | The acute inflammatory response to lipopolysaccharide in CNS parenchyma differs from that in other b... | 1992 | Fundamental biology study |
| 58 | Tolerance of rat small intestine to localized single dose and fractionated irradiation | 1992 | Fundamental biology study |
| 59 | Tumour occurrence and recurrence | 1992 | Does not meet search criteria |
| 60 | Brain hydroxyl radical generation in acute experimental head injury | 1993 | Fundamental biology study |
| 61 | Changes of vinculin and extracellular matrix components following blunt trauma to rat skeletal muscl... | 1993 | Fundamental biology study |
| 62 | Contrasting effects of immunosuppression on herpes simplex virus type I (HSV I) induced central nerv... | 1993 | Fundamental biology study |
| 63 | Early changes in second messenger but not receptor binding sites after acute subdural hematoma: an i... | 1993 | Fundamental biology study |
| 64 | Effect of neonatal capsaicin treatment on neurogenic pulmonary edema from fluid-percussion brain inj... | 1993 | Fundamental biology study |
| 65 | Effect of radiation therapy and Photofrin on tissue response in a rat model | 1993 | Existing clinical therapy at publication |
| 66 | Evans blue extravasation in rat dental pulp and oral tissues induced by electrical stimulation of th... | 1993 | Fundamental biology study |
| 67 | Extreme hypothermia induced by a synergism of acute limbic seizures, physical restraint, and aceprom... | 1993 | Fundamental biology study |
| 68 | Increased negatively of interstitial fluid pressure in rat skin contributes to the edema formation i... | 1993 | Fundamental biology study |
| 69 | Inhibition of wound contraction by topical antimicrobials | 1993 | Existing clinical therapy at publication |
| 70 | Laser induced wounds and scarring modified by antiinflammatory drugs: a murine model | 1993 | Existing clinical therapy at publication |
| 71 | Neutrophils, differentiated macrophages, and monocyte/macrophage antigen presenting cells infiltrate... | 1993 | Fundamental biology study |
| 72 | Rubrospinal neurons and retrograde transport of fluoro-gold in acute spinal cord injury--a dose-resp... | 1993 | Fundamental biology study |
| 73 | Snakebite-induced acute renal failure: an experimental model | 1993 | Fundamental biology study |
| 74 | Studies on the migration of micronucleated erythrocytes from bone marrow to the peripheral blood in ... | 1993 | Fundamental biology study |
| 75 | Tetanus prophylaxis following ocular injuries | 1993 | Existing clinical therapy at publication |
| 76 | The biokinetics of plutonium-239 and americium-241 in the rat after subcutaneous deposition of conta... | 1993 | Fundamental biology study |
| 77 | Acute subdural hematoma: is the blood itself toxic? | 1994 | Fundamental biology study |
| 78 | Angiotensin converting enzyme inhibition and coronary artery disease | 1994 | Review/not original research |
| 79 | Arterial blood pressure immediately after thermal injury: the role of anesthesia | 1994 | Fundamental biology study |
| 80 | Bacterial translocation in burned mice after administration of various diets including fiber- and gl... | 1994 | Fundamental biology study |
| 81 | Comparison of diffusion-weighted MRI with changes in cell volume in a rat model of brain injury | 1994 | Fundamental biology study |
| 82 | Dynorphin A-induced rat spinal cord injury: evidence for excitatory amino acid involvement in a phar... | 1994 | Fundamental biology study |
| 83 | Effects of neutropenia on edema, histology, and cerebral blood flow after traumatic brain injury in ... | 1994 | Fundamental biology study |
| 84 | Electrophysiological studies on light damage in the mouse retina after sodium iodate injection | 1994 | Fundamental biology study |
| 85 | Endogenous phosphorylation of a 61,000 dalton hippocampal protein increases following traumatic brai... | 1994 | Fundamental biology study |
| 86 | Expression of growth factors and receptors during specific phases in regenerating urothelium after a... | 1994 | Fundamental biology study |
| 87 | Expression of growth factors in early wound healing in rat skin | 1994 | Fundamental biology study |
| 88 | Expression of murine VCAM-1 in vitro and in different models of inflammation in vivo: correlation wi... | 1994 | Fundamental biology study |
| 89 | Influence of capsaicin-sensitive afferent fibers on acetic acid-induced chronic gastric ulcers in ra... | 1994 | Fundamental biology study |
| 90 | Pneumonia in the surgical intensive care unit. Immunologic keys to the silent epidemic | 1994 | Does not meet search criteria |
| 91 | Pretreatment of kidney allografts with monoclonal antibodies to CD45: results of a multicentre study... | 1994 | Does not meet search criteria |
| 92 | Relationship between ultraviolet radiation-induced immunosuppression and carcinogenesis | 1994 | Review/not original research |
| 93 | Response of rat skin to boron neutron capture therapy with p-boronophenylalanine or borocaptate sodi... | 1994 | Existing clinical therapy at publication |
| 94 | Role of early enteral feeding and acute starvation on postburn bacterial translocation and host defe... | 1994 | Fundamental biology study |
| 95 | Therapeutic effects of water and milk for acute alkali injury of the esophagus | 1994 | Fundamental biology study |
| 96 | A perpetual cascade of cytokines postirradiation leads to pulmonary fibrosis | 1995 | Fundamental biology study |
| 97 | Acute and subacute inhalation toxicity of diborane in male ICR mice | 1995 | Fundamental biology study |
| 98 | Acute cocaine administration alters posttraumatic blood pressure and cerebral blood flow in rats | 1995 | Fundamental biology study |
| 99 | Blood-brain barrier disturbance following localized hyperthermia in rats | 1995 | Fundamental biology study |
| 100 | Chemical-induced vasculature injury. Summary of the symposium presented at the 32nd annual meeting o... | 1995 | Does not meet search criteria |
| 101 | Chronic aspirin treatment affects collagen deposition in non-infarcted myocardium during remodeling ... | 1995 | Existing clinical therapy at publication |
| 102 | Detection of platelet-derived growth factor (PDGF)-AA in actively healing human wounds treated with ... | 1995 | Does not meet search criteria |
| 103 | Dysfunction of cGMP-mediated pulmonary vasorelaxation in endotoxin-induced acute lung injury | 1995 | Fundamental biology study |
| 104 | Effective treatment for acute alkali injury to the esophagus using weak-acid neutralization therapy:... | 1995 | Fundamental biology study |
| 105 | Effects of acute ethanol intoxication on experimental brain injury in the rat: neurobehavioral and p... | 1995 | Fundamental biology study |
| 106 | Effects of high-dose vitamin C administration on bacterial translocation and lung neutrophil sequest... | 1995 | Existing clinical therapy at publication |
| 107 | Epidermal growth factor and basic fibroblast growth factor are induced in guinea-pig tympanic membra... | 1995 | Fundamental biology study |
| 108 | Episodic hypertension due to autonomic dysreflexia in acute and chronic spinal cord-injured rats | 1995 | Fundamental biology study |
| 109 | Expression of murine VCAM-1 in vitro and in different models of inflammation in vivo: correlation wi... | 1995 | Fundamental biology study |
| 110 | Expression of the beta 6 integrin subunit in development, neoplasia and tissue repair suggests a rol... | 1995 | Does not meet search criteria |
| 111 | Expression of transforming growth factor-beta 1 in mouse skin during the acute phase of radiation da... | 1995 | Fundamental biology study |
| 112 | Hydroxyl radical production and lung injury in the rat following silica or titanium dioxide instilla... | 1995 | Fundamental biology study |
| 113 | Immunoglobulin M synthesis after burn injury: the effects of chronic ethanol on postinjury synthesis | 1995 | Fundamental biology study |
| 114 | Increased immunoreactive rat lung ICAM-1 in oleic acid-induced lung injury | 1995 | Fundamental biology study |
| 115 | Inhibition of cellular ras prevents smooth muscle cell proliferation after vascular injury in vivo | 1995 | Fundamental biology study |
| 116 | Investigation of lung tumour induction in BALB/cJ mice following paternal X-irradiation | 1995 | Fundamental biology study |
| 117 | Myocardial infarction and the myocyte IGF1 autocrine system | 1995 | Fundamental biology study |
| 118 | Noninvasive follow-up of tissue encapsulation of foreign materials. Are magnetic resonance imaging a... | 1995 | Fundamental biology study |
| 119 | Ocular resurfacing and alloepithelial rejection in a murine keratoepithelioplasty model. | 1995 | Fundamental biology study |
| 120 | Plasticity of airway cell proliferation and gene expression after acute naphthalene injury | 1995 | Fundamental biology study |
| 121 | Production of chronic compression of the cauda equina in rats for use in studies of lumbar spinal ca... | 1995 | Fundamental biology study |
| 122 | The rationale for glutamate antagonists in the treatment of traumatic brain injury | 1995 | Review/not original research |
| 123 | The use of ultrasound as an enhancer for transcutaneous drug delivery: phonophoresis | 1995 | Review/not original research |
| 124 | Transforming growth factor beta 1, extracellular matrix, and inflammatory cells in wound repair usin... | 1995 | Fundamental biology study |
| 125 | c-myc in vasculoproliferative disease | 1995 | Fundamental biology study |
| 126 | A ferret model of electrical-induction of arterial thrombosis that is sensitive to aspirin | 1996 | Does not meet search criteria |
| 127 | Action of sensory neurons in an experimental at colitis model of injury and repair | 1996 | Fundamental biology study |
| 128 | Acute epithelial injury in the rat small intestine in vivo is associated with expanded expression of... | 1996 | Fundamental biology study |
| 129 | Alterations in CNS gene expression in a rodent model of moderate traumatic brain injury complicated ... | 1996 | Fundamental biology study |
| 130 | Biogenesis of hepatic acute-phase response to trauma | 1996 | Does not meet search criteria |
| 131 | Changes in pharmacological sensitivity of the spinal cord to potassium channel blockers following ac... | 1996 | Fundamental biology study |
| 132 | Characterization of a murine model of acute lung injury (ALI): a prominent role for interleukin-1 | 1996 | Fundamental biology study |
| 133 | Development and use of an animal model for investigations on rotator cuff disease | 1996 | Fundamental biology study |
| 134 | Effect of high-dose pentoxifylline on acute radiation-induced lung toxicity in a rat lung perfusion ... | 1996 | Fundamental biology study |
| 135 | Enhanced inflammation and immunosuppression by ultraviolet radiation in xeroderma pigmentosum group ... | 1996 | Fundamental biology study |
| 136 | Excitotoxicity in the lung: N-methyl-D-aspartate-induced, nitric oxide-dependent, pulmonary edema is... | 1996 | Does not meet search criteria |
| 137 | Experimental brain injury induces differential expression of tumor necrosis factor-alpha mRNA in the... | 1996 | Fundamental biology study |
| 138 | Healing of tympanic membrane after myringotomy during Streptococcus pneumoniae otitis media. An otom... | 1996 | Fundamental biology study |
| 139 | Impact of receptor downregulation on clearance of two human EGFs with different receptor binding act... | 1996 | Fundamental biology study |
| 140 | Impairment of endothelial-dependent pulmonary vasorelaxation after mesenteric ischemia/reperfusion | 1996 | Fundamental biology study |
| 141 | In vivo inflammatory response to silicone elastomer particulate debris | 1996 | Fundamental biology study |
| 142 | Influence of fraction size on the development of late radiation enteropathy. An experimental study i... | 1996 | Fundamental biology study |
| 143 | Influence of steroid injection on ligament healing in the rat | 1996 | Existing clinical therapy at publication |
| 144 | Kinetics of cytokine expression during healing of acute colitis in mice | 1996 | Fundamental biology study |
| 145 | Light damage in rod outer segments: the effects of fixation on ultrastructural alterations | 1996 | Fundamental biology study |
| 146 | Mast cell clusters in pars tensa membranae tympani in acute otitis media: a possible role in perfora... | 1996 | Fundamental biology study |
| 147 | Neural and endocrine circuits mediating inhibition of bradykinin-induced plasma extravasation by sub... | 1996 | Fundamental biology study |
| 148 | Neural and endocrine circuits mediating inhibition of bradykinin-induced plasma extravasation by sub... | 1996 | Fundamental biology study |
| 149 | Neuroprotection by propofol in acute mechanical injury: role of GABAergic inhibition | 1996 | Does not meet search criteria |
| 150 | Role of radioadaptation on radiation-induced thymic lymphoma in mice | 1996 | Fundamental biology study |
| 151 | Temporal and spatial characterization of neuronal injury following lateral fluid-percussion brain in... | 1996 | Fundamental biology study |
| 152 | The effect of a muscle wrap on nerve healing in a rat model | 1996 | Fundamental biology study |
| 153 | The role of blood flow in chronic duodenal ulcer | 1996 | Fundamental biology study |
| 154 | The role of spinal cord activation before neurectomy in the development of autotomy | 1996 | Fundamental biology study |
| 155 | Thrombospondin receptor (CD36) expression of human keratinocytes during wound healing in a SCID mous... | 1996 | Fundamental biology study |
| 156 | Tissue reactions to biodegradable and non-degradable membranes placed transcutaneously in rats, obse... | 1996 | Fundamental biology study |
| 157 | Traumatic neuroprotection with inhibitors of nitric oxide and ADP-ribosylation | 1996 | Fundamental biology study |
| 158 | Vascular cell adhesion molecule-1 mRNA is expressed in immune-mediated and ischemic injury of the ra... | 1996 | Fundamental biology study |
| 159 | Visceral ischemia-reperfusion injury promotes tumor necrosis factor (TNF) and interleukin-1 (IL-1) d... | 1996 | Fundamental biology study |
| 160 | mu-calpain activation and calpain-mediated cytoskeletal proteolysis following traumatic brain injury | 1996 | Fundamental biology study |
| 161 | A new model of acute compressive spinal cord injury in vitro | 1997 | Does not meet search criteria |
| 162 | Acute erosions of the gastric mucosa in burned rats: effect of sucralfate | 1997 | Existing clinical therapy at publication |
| 163 | Alloxan diabetes abolishes the increased negativity of interstitial fluid pressure in rat trachea in... | 1997 | Fundamental biology study |
| 164 | Alveolar neutrophils in endotoxin-induced and bacteria-induced acute lung injury in rats | 1997 | Fundamental biology study |
| 165 | An experimental study of the neurogenic and the immunological contribution to "tennis elbow" in rats | 1997 | Does not meet search criteria |
| 166 | Analytical performance and clinical utility of a sensitive immunoassay for determination of human ca... | 1997 | Does not meet search criteria |
| 167 | Bcl-x expression influences keratinocyte cell survival but not terminal differentiation | 1997 | Fundamental biology study |
| 168 | Biomechanical and biochemical study of a standardized wound healing model | 1997 | Fundamental biology study |
| 169 | Blockade of basic fibroblast growth factor retards recovery from motor cortex injury in rats | 1997 | Fundamental biology study |
| 170 | Cell proliferation contributes to PNEC hyperplasia after acute airway injury | 1997 | Fundamental biology study |
| 171 | Changes in cochlear function after double-membrane rupture in the guinea pig | 1997 | Fundamental biology study |
| 172 | Delayed molecular responses to brain irradiation | 1997 | Fundamental biology study |
| 173 | Diagnosis and follow-up of muscle injuries by means of plain and contrast-enhanced MRT: experimental... | 1997 | Non-English article |
| 174 | Differential activation of the Stat signaling pathway in the liver after burn injury | 1997 | Fundamental biology study |
| 175 | Diminished inflammation and nociceptive pain with preservation of neuropathic pain in mice with a ta... | 1997 | Fundamental biology study |
| 176 | Distribution of epidermal growth factor receptor and ligands during bronchiolar epithelial repair fr... | 1997 | Fundamental biology study |
| 177 | Effective management of microbial contamination in cultured skin substitutes after grafting to athym... | 1997 | Fundamental biology study |
| 178 | Effects of acute ethanol exposure on cellular immune responses in a murine model of thermal injury | 1997 | Fundamental biology study |
| 179 | Effects of ageing and X-irradiation on the diurnal rhythm of mouse urinary bladder capacity | 1997 | Fundamental biology study |
| 180 | Estrogen accelerates cutaneous wound healing associated with an increase in TGF-beta1 levels | 1997 | Does not meet search criteria |
| 181 | Estrogens attenuate neuronal injury due to hemoglobin, chemical hypoxia, and excitatory amino acids ... | 1997 | Fundamental biology study |
| 182 | Evaluation of the temporal evolution of acute spinal cord injury | 1997 | Fundamental biology study |
| 183 | Experimental study on the treatment of smoke inhalation injury with lung lavage and exogenous pulmon... | 1997 | Non-English article |
| 184 | Expression of endothelial adhesion molecules and recruitment of neutrophils after traumatic brain in... | 1997 | Fundamental biology study |
| 185 | Expression of transforming growth factor-beta receptors during hyperoxia-induced lung injury and rep... | 1997 | Fundamental biology study |
| 186 | Genetic modeling of susceptibility to nitrogen dioxide-induced lung injury in mice | 1997 | Fundamental biology study |
| 187 | High-resolution 1H NMR spectroscopy following experimental brain trauma | 1997 | Fundamental biology study |
| 188 | Importance of nitric oxide and capsaicin-sensitive afferent nerves in healing of stress lesions indu... | 1997 | Fundamental biology study |
| 189 | In vivo biocompatibility and biostability of modified polyurethanes | 1997 | Fundamental biology study |
| 190 | Increased transforming growth factor beta (TGF-beta) immunoreactivity is independently associated wi... | 1997 | Fundamental biology study |
| 191 | Induction of cyclooxygenase 2 in gastric mucosal lesions and its inhibition by the specific antagoni... | 1997 | Fundamental biology study |
| 192 | Inflammatory gene expression in cerebral ischemia and trauma. Potential new therapeutic targets | 1997 | Review/not original research |
| 193 | Interleukin-6-dependent and -independent regulation of the human C-reactive protein gene | 1997 | Fundamental biology study |
| 194 | Leukotrienes are indicated as mediators of hyperoxia-inhibited alveolarization in newborn rats | 1997 | Fundamental biology study |
| 195 | Murine laminin alpha3A and alpha3B isoform chains are generated by usage of two promoters and altern... | 1997 | Does not meet search criteria |
| 196 | Neurohormonal mechanisms of cigarette smoke-induced duodenal mucosal bicarbonate secretion in the ra... | 1997 | Fundamental biology study |
| 197 | Paradoxical effects of acute ethanolism in experimental brain injury | 1997 | Fundamental biology study |
| 198 | Pharmacologic approaches to protection against radiation-induced lethality and other damage | 1997 | Review/not original research |
| 199 | Production and activation of hepatocyte growth factor during the healing of rat gastric ulcers | 1997 | Fundamental biology study |
| 200 | Reduced engraftment and wound closure of cryopreserved cultured skin substitutes grafted to athymic ... | 1997 | Fundamental biology study |

**Note:** This table shows the first 200 of 1194 excluded studies. The complete dataset including all 1194 excluded studies with full metadata (Title, DOI, Animal model, Year, Journal, Exclusion code) is available in the supplementary Excel file deposited in the data repository.

## Exclusion Criteria Key

**1.** Unsuccessful/negative preclinical study

**2.** Does not meet search criteria

**3.** Predatory journal

**4.** Existing clinical therapy at publication

**5.** Fundamental biology study

**6.** Not a therapy R&D study

# **Section S6 - Sensitivity Analyses Results**

## 1. Database Coverage Sensitivity

To assess potential impact of single-database searching, we conducted targeted Embase searches for three exemplar therapies with high preclinical publication volume:

| **Therapy** | **PubMed hits** | **Embase-only hits** | **Additional eligible** |
| --- | --- | --- | --- |
| Progesterone (TBI) | 47 | 3 | 0 |
| Erythropoietin (CNS) | 38 | 5 | 0 |
| Minocycline (SCI) | 29 | 2 | 0 |

Conclusion: No additional eligible studies identified through Embase sensitivity check.

## 2. Classification Sensitivity

To assess robustness of regulatory classification, we recalculated translation rates under alternative classification rules:

| **Classification Rule** | **n successful** | **Rate (95% CI)** |
| --- | --- | --- |
| Primary analysis (TRUE SUCCESS only) | 3 | 3.6% (1.2-10.1%) |
| Including SUCCESS WITH CAVEATS | 7 | 8.4% (4.1-16.4%) |
| Including REGIONAL ONLY | 12 | 14.5% (8.4-23.8%) |
| Including DIFFERENT INDICATION | 41 | 49.4% (38.9-59.9%) |
| Any clinical trial entry (headline) | 251/1257 | 20.0% (17.9-22.3%) |

## 3. Timeframe Sensitivity

Translation rates by publication period:

| **Period** | **Studies** | **Translated** | **Rate** |
| --- | --- | --- | --- |
| 1990-1999 | 412 | 71 | 17.2% |
| 2000-2010 | 845 | 180 | 21.3% |

No significant difference in translation rates between earlier and later publication periods (chi-square p=0.09).

## 4. Species Sensitivity

| **Species** | **Studies** | **Translated** | **Rate** |
| --- | --- | --- | --- |
| Rat | 882 | 178 | 20.2% |
| Mouse | 284 | 56 | 19.7% |
| Other | 91 | 17 | 18.7% |

Translation rates were consistent across species, suggesting findings are not driven by species-specific effects.

# **Table S1**

## Characteristics of 1,257 Included Studies

### A. Species Distribution

| **Species** | **n** | **%** |
| --- | --- | --- |
| Rat | 882 | 70.2 |
| Mouse | 284 | 22.6 |
| Guinea pig | 32 | 2.5 |
| Other* | 59 | 4.7 |
| **Total** | **1,257** | **100.0** |

*Other includes: hamsters, rabbits, chinchillas, multi-species studies, diabetic model variants, and studies with unspecified species.

### B. Publication Year Distribution

| **Year** | **n** | **%** |
| --- | --- | --- |
| 1990 | 10 | 0.8 |
| 1991 | 8 | 0.6 |
| 1992 | 12 | 1.1 |
| 1993 | 16 | 1.3 |
| 1994 | 16 | 1.3 |
| 1995 | 18 | 1.4 |
| 1996 | 23 | 1.8 |
| 1997 | 34 | 2.7 |
| 1998 | 33 | 2.6 |
| 1999 | 57 | 4.5 |
| 2000 | 53 | 4.2 |
| 2001 | 58 | 4.6 |
| 2002 | 59 | 4.7 |
| 2003 | 71 | 5.6 |
| 2004 | 86 | 6.8 |
| 2005 | 80 | 6.4 |
| 2006 | 93 | 7.4 |
| 2007 | 122 | 9.7 |
| 2008 | 89 | 7.1 |
| 2009 | 133 | 10.6 |
| 2010 | 130 | 10.3 |
| Not specified | 56 | 4.5 |
| **Total** | **1,257** | **100.0** |

### C. Most Frequently Studied Therapies (≥3 studies, comprehensive therapy grouping including all naming variants)

| **Therapy** | **n** | **%** |
| --- | --- | --- |
| Erythropoietin | 17 | 1.4 |
| Hypothermia | 16 | 1.3 |
| Progesterone | 12 | 1.0 |
| Minocycline | 7 | 0.6 |
| Hyperbaric oxygen | 6 | 0.5 |
| Magnesium sulphate | 5 | 0.4 |
| Melatonin | 5 | 0.4 |
| Curcumin | 5 | 0.4 |
| Polyethylene glycol | 5 | 0.4 |
| Hypertonic saline | 4 | 0.3 |
| Resveratrol | 3 | 0.2 |
| Olfactory ensheathing cells | 3 | 0.2 |
| MK-801 (Dizocilpine) | 3 | 0.2 |
| Simvastatin | 3 | 0.2 |
| Nicotinamide | 3 | 0.2 |

### D. Translation to Clinical Trial

| **Status** | **n** | **%** |
| --- | --- | --- |
| Yes (any translation) | 251 | 20.0 |
| No | 1,006 | 80.0 |

# **Table S2 - Complete Therapy Classification Database (100 therapies)**

| **Therapy** | **Preclinical Indication** | **Regulatory Status** | **Approved Indication** | **Notes** |
| --- | --- | --- | --- | --- |
| Palifermin (KGF) | Oral mucositis | TRUE SUCCESS | Oral mucositis in haematological maligna | FDA approved 2004 |
| rhBMP-2 | Fracture healing | TRUE SUCCESS | Spinal fusion, tibial fractures | FDA approved for bone healing |
| Recombinant human GH | Burns/catabolism | TRUE SUCCESS | Severe burns catabolism | FDA approved for burns |
| Mannitol | Cerebral oedema | SUCCESS WITH CAVEATS | Cerebral oedema | Approved ~1960s |
| Pantoprazole | Gastric ulcer | SUCCESS WITH CAVEATS | GORD, gastric/duodenal ulcers | Member of approved class |
| Beractant (Survanta) | Lung injury/RDS | SUCCESS WITH CAVEATS | Neonatal respiratory distress syndrome | Biological replacement therapy |
| Autologous keratinocytes | Burns/skin wounds | SUCCESS WITH CAVEATS | Burns, chronic wounds | Epicel FDA approved |
| 4-aminopyridine (Dalfampridine | Spinal cord injury | APPROVED - DIFFERENT INDICATION | Walking impairment in MS | FDA 2010 for MS; SCI trials inconclusive |
| Edaravone (Radicava) | Radiation/CNS injury | APPROVED - DIFFERENT INDICATION | ALS (2017) | Not approved for radiation or acute CNS injury |
| Filgrastim (G-CSF) | Myocardial infarction | APPROVED - DIFFERENT INDICATION | Neutropenia | Failed in MI trials |
| Etanercept (Enbrel) | Lung injury | APPROVED - DIFFERENT INDICATION | Rheumatoid arthritis, psoriasis | Not approved for lung injury |
| Cyclosporin A | Traumatic brain injury | APPROVED - DIFFERENT INDICATION | Transplant rejection | NeuroSTAT trial failed for TBI |
| Minocycline | Spinal cord/brain injury | APPROVED - DIFFERENT INDICATION | Bacterial infections, acne | Failed in multiple CNS injury trials |
| Riluzole | Spinal cord injury | APPROVED - DIFFERENT INDICATION | ALS | SCI trials ongoing/inconclusive |
| Valproate | Traumatic brain injury | APPROVED - DIFFERENT INDICATION | Epilepsy, bipolar disorder | TBI trials inconclusive |
| Celecoxib | Radiotherapy/skin cancer | APPROVED - DIFFERENT INDICATION | Arthritis, pain | Mixed results in cancer prevention trials |
| Simvastatin | Brain/CNS injury | APPROVED - DIFFERENT INDICATION | Hypercholesterolaemia | Failed in CNS injury trials |
| Atorvastatin | Brain injury | APPROVED - DIFFERENT INDICATION | Hypercholesterolaemia | Failed in TBI trials |
| Lovastatin | Radiotherapy protection | APPROVED - DIFFERENT INDICATION | Hypercholesterolaemia | Not approved for radioprotection |
| Memantine | Neuropathic pain | APPROVED - DIFFERENT INDICATION | Alzheimer's disease | Limited efficacy in pain |
| Levetiracetam (Keppra) | Head injury | APPROVED - DIFFERENT INDICATION | Epilepsy | Off-label use; not specifically approved for TBI |
| Enoxaparin | Traumatic brain injury | APPROVED - DIFFERENT INDICATION | DVT prophylaxis | Not approved for TBI treatment |
| Mifepristone (RU38486) | PTSD | APPROVED - DIFFERENT INDICATION | Medical abortion | Not approved for PTSD |
| Octreotide | Radiation enteropathy | APPROVED - DIFFERENT INDICATION | Acromegaly, carcinoid tumours | Limited evidence for radiation GI injury |
| Propranolol | Wound healing | APPROVED - DIFFERENT INDICATION | Hypertension, anxiety, tremor | Not approved for wound healing |
| Oxandrolone | Spinal cord injury | APPROVED - DIFFERENT INDICATION | Muscle wasting, burns | Not approved for SCI recovery |
| Sucralfate | Radiotherapy mucositis | APPROVED - DIFFERENT INDICATION | Peptic ulcer | Mixed results in radiation mucositis |
| Pentoxifylline | Radiotherapy injury | APPROVED - DIFFERENT INDICATION | Peripheral vascular disease | Some use in radiation fibrosis; limited evidence |
| Dobutamine | Lung injury | APPROVED - DIFFERENT INDICATION | Cardiac support | Not approved for acute lung injury |
| Melatonin | Wound healing | APPROVED - DIFFERENT INDICATION | Sleep disorders (OTC) | Not approved as drug for wound healing |
| Insulin glargine | Burns/metabolic | APPROVED - DIFFERENT INDICATION | Diabetes mellitus | Not approved for burns |
| N-acetylcysteine | Lung/muscle injury | APPROVED - DIFFERENT INDICATION | Paracetamol overdose, mucolytic | Not approved for acute injury |
| Estradiol/Oestrogen | CNS/vascular injury | APPROVED - DIFFERENT INDICATION | Hormone replacement therapy | Not approved for acute injury |
| Growth hormone | Various injuries | APPROVED - DIFFERENT INDICATION | Growth hormone deficiency | Distinct from rhGH (TRUE SUCCESS for burns); GH for non-burn acute injuries did not achieve approval for tested indications |
| NNZ-2566 (Trofinetide) | Brain injury | APPROVED - DIFFERENT INDICATION | Rett syndrome (FDA 2023) | Failed for TBI; approved for Rett syndrome |
| Lidocaine | Neuropathic pain | APPROVED - DIFFERENT INDICATION | Local anaesthesia | Not approved for intrathecal chronic use |
| Progesterone | Traumatic brain injury | FAILED |  | ProTECT III and SYNAPSE both failed 2014; >2000 pa |
| Erythropoietin | Brain/spinal cord injury | FAILED |  | Multiple large CNS injury trials failed |
| Magnesium sulphate | Brain trauma/stroke | FAILED |  | IMAGES trial negative for stroke; TBI trials negat |
| Tirilazad (U-74006F) | CNS injury | FAILED |  | Failed in multiple stroke and TBI trials |
| Repinotan (BAY x3702) | CNS injury/stroke | FAILED |  | Phase III stroke trial terminated for futility |
| Clazosentan | Cerebral vasospasm | FAILED |  | CONSCIOUS-2 and CONSCIOUS-3 trials failed |
| Cethrin (BA-210) | Spinal cord injury | FAILED |  | Did not meet primary endpoints |
| Velafermin (rhFGF-20) | Oral mucositis | FAILED |  | Development discontinued |
| Depelestat (EPI-hNE-4) | ARDS/lung injury | FAILED |  | Failed in ARDS trials |
| Carisbamate | Post-traumatic epilepsy | FAILED |  | Development discontinued after Phase III failure |
| OT-551/TEMPOL | Retinal/radiation injury | FAILED |  | Phase II AMD trial failed |
| Delucemine (NPS 1506) | Brain trauma | FAILED |  | Development discontinued |
| d-Amphetamine | Stroke recovery | FAILED |  | AFFINITY trial negative |
| Paclitaxel polyglumex | Radiotherapy | FAILED |  | Development discontinued |
| Hypertonic hydroxyethyl starch | Shock/trauma | FAILED |  | Safety concerns; EMA restrictions |
| Mesenchymal stem cells | Multiple injury types | NOT APPROVED |  | No regulatory approval for injury indications |
| Bone marrow stromal cells | Spinal cord injury | NOT APPROVED |  | Still in early clinical trials |
| Schwann cell transplant | Spinal cord injury | NOT APPROVED |  | Still experimental |
| Olfactory ensheathing cells | Spinal cord injury | NOT APPROVED |  | Still experimental; mixed results |
| Human umbilical cord MSCs | Spinal cord injury | NOT APPROVED |  | No regulatory approval |
| CD34+ cells | Heart disease | NOT APPROVED |  | Still in clinical trials |
| Autologous EPCs | Cardiovascular disease | NOT APPROVED |  | Still experimental |
| Neural stem/progenitor cells | Spinal cord injury | NOT APPROVED |  | Still in early trials |
| Choroid plexus xenotransplant | CNS injury | NOT APPROVED |  | Limited clinical development |
| Hepatocyte growth factor | Spinal cord injury | NOT APPROVED |  | Still in clinical trials |
| Resveratrol | Brain injury | NOT APPROVED |  | Supplement; bioavailability issues |
| Curcumin | Radiotherapy injury | NOT APPROVED |  | Supplement; bioavailability issues |
| Genistein | Radiation injury | NOT APPROVED |  | Still in development |
| Ascorbic acid (Vitamin C) | Radiotherapy | NOT APPROVED |  | OTC supplement |
| Creatine | Spinal cord injury | NOT APPROVED |  | Supplement; not approved as drug |
| Blackberry extract | Skin cancer/UV damage | NOT APPROVED |  | Early development |
| Flavonoids | Inflammation | NOT APPROVED |  | Supplements; not approved as drugs |
| Selenium | Spinal cord injury | NOT APPROVED |  | Supplement; not approved for SCI |
| L-NAME (NOS inhibitor) | Spinal cord injury | NOT APPROVED |  | Never advanced to approval |
| VAS203 (NOS inhibitor) | Brain injury | NOT APPROVED |  | Phase III trials planned/ongoing |
| Entolimod (CBLB502) | Radiation injury | NOT APPROVED |  | Still in development |
| ST266/ACCS | Wound healing | NOT APPROVED |  | Still in development |
| Recombinant plasma gelsolin | Lung injury | NOT APPROVED |  | Still in development |
| Hypertonic saline | Brain injury | NOT APPROVED |  | Used off-label; not specifically approved for TBI |
| KBPA101 antibody | Infection | NOT APPROVED |  | Limited public information |
| Alpha-tocopherol (Vitamin E) | Radiation/skin | NOT APPROVED |  | Supplement; not approved as drug |
| Platelet-rich plasma | Muscle injury | NOT APPROVED |  | Widely used but not FDA approved |
| Sivelestat (Elaspol) | ARDS/lung injury | REGIONAL ONLY | ARDS (Japan, Korea only) | Not approved in US or EU |
| Anti-NGF (Tanezumab) | Pain | REGIONAL ONLY | Osteoarthritis pain (EU 2022) | US approval pending; safety concerns |
| Polaprezinc | Gastric injury | REGIONAL ONLY | Gastric ulcer (Japan) | Not approved in US/EU |
| Geranyl-geranyl-acetone | Gastric/various | REGIONAL ONLY | Gastric ulcer (Japan) | Not approved in US/EU |
| Ketanserin | Wound healing | REGIONAL ONLY | Hypertension (some countries) | Not approved in US |
| Hypothermia | TBI/Perinatal asphyxia | NON-DRUG | Neonatal HIE (standard of care) | Physical therapy, not a drug |
| Hyperbaric oxygen | Brain injury | NON-DRUG | Decompression sickness, wounds | Physical therapy |
| Acupuncture | Spinal cord injury | NON-DRUG |  | Evidence mixed; not a drug |
| Exercise therapy | Traumatic brain injury | NON-DRUG |  | Rehabilitation approach |
| Cryotherapy | Musculoskeletal trauma | NON-DRUG |  | Standard practice |
| Intermittent hypoxia | Spinal cord injury | NON-DRUG |  | Physical intervention |
| Ischaemic conditioning | Various | NON-DRUG |  | Physical intervention |
| Electrical stimulation | Spinal cord injury | DEVICE | Various indications | Medical device, not drug |
| Transcorneal electrical stim | Optic nerve injury | DEVICE |  | Still in development |
| ESWT (Shock wave) | Burn/musculoskeletal | DEVICE | Musculoskeletal conditions | Not specifically for burns |
| Aquacel hydrofibre | Wound healing | DEVICE | Wound care | Medical device, not drug |
| Permacol | Soft tissue repair | DEVICE | Hernia repair, soft tissue | Medical device, not drug |
| Small intestinal submucosa | Wound healing | DEVICE | Wound care | Medical device, not drug |
| Ultrasonic scalpel | Surgery | DEVICE | Surgical procedures | Medical device, not drug |
| Gore-Tex PTFE | Abdominal wall | DEVICE | Hernia repair | Medical device, not drug |
| Er:YAG laser | Skin resurfacing | DEVICE | Dermatological procedures | Medical device, not drug |
| Aquamid | Soft tissue | DEVICE | Dermal filler (EU) | Not FDA approved |

# **Table S3 - 15 Clinical Trial Failures with Trial Citations**

| **Therapy** | **Indication** | **Key Trial(s)** | **Outcome** | **Citation** |
| --- | --- | --- | --- | --- |
| Progesterone | TBI | ProTECT III, SyNAPSe | No benefit vs placebo (2014) | Wright NEJM 2014; Skolnick NEJM 2014 |
| Erythropoietin | TBI/SCI | EPO-TBI | No benefit, trend to harm | Nichol JAMA 2015 |
| Tirilazad (U-74006F) | Stroke/TBI | RANTTAS, HIT I-IV | Failed all Phase III | Lees Lancet 2000 |
| Magnesium sulphate | Brain trauma/stroke | IMAGES | No benefit | Muir Lancet 2004 |
| Repinotan (BAY x3702) | CNS injury/stroke | Phase III stroke trial | Terminated for futility | Teal Stroke 2009 |
| Clazosentan | Cerebral vasospasm | CONSCIOUS-2, CONSCIOUS-3 | Failed primary endpoint | Macdonald Lancet Neurol 2011 |
| Cethrin (BA-210) | Spinal cord injury | Phase I/IIa | Did not meet primary endpoints | Fehlings J Neurotrauma 2011 |
| Velafermin (rhFGF-20) | Oral mucositis | Phase II | Development discontinued | Freytes Oral Oncol 2004 |
| Depelestat (EPI-hNE-4) | ARDS/lung injury | Phase II | Failed in ARDS | Zeiher Am J Respir Crit Care Med 2004 |
| Carisbamate | Post-traumatic epilepsy | Phase III | Development discontinued | Faught Epilepsia 2014 |
| OT-551/TEMPOL | Retinal/radiation injury | Phase II (AMD) | No benefit | Wong Am J Ophthalmol 2010 |
| Delucemine (NPS 1506) | Brain trauma | Phase II | Development discontinued | Unpublished |
| d-Amphetamine | Stroke recovery | AFFINITY | No benefit | AFFINITY Trial Lancet Neurol 2020 |
| Paclitaxel polyglumex | Radiotherapy | Phase III | Development discontinued | Langer J Clin Oncol 2008 |
| Hypertonic hydroxyethyl starch | Shock/trauma | Multiple RCTs | Safety concerns; EMA restrictions | Perner NEJM 2012 |

# **Table S4 - Post-market Safety Data Sources and Detailed Findings**

| **Drug** | **Data Sources** | **Key Findings** |
| --- | --- | --- |
| rhBMP-2 | FDA FAERS; FDA PHN 2008; Yale/YODA 2013; Senate Report 2012 | AE rates 10-50× higher; cancer RR 3.45; ~6,000 lawsuits; >$100M settlements |
| rhGH (burns) | Takala NEJM 1999; FDA label | Mortality 44% vs 18% (RR 2.4); trial stopped; contraindicated in critically ill |
| Pantoprazole | FDA DSC 2010-2014; JAMA studies | 5 FDA warnings; CKD +20-50%; fractures +30%; 18,600 lawsuits; $590M settlements |
| Palifermin | FDA label; Trial reports | Trial stopped - worsened mucositis; narrowed to autologous only |
| Mannitol | FDA label; NCS 2020; BTF 2017 | Renal failure risk; paradoxical oedema; NCS "unable to recommend" |
| Beractant | FDA label; Post-marketing | Sepsis 20.7% vs 16.1%; relatively clean profile |
| Epicel | FDA HDE docs; 2022 update | "Effectiveness not demonstrated"; 13 SCC cases |

# **Table S5 - 29 Drugs Approved for Different Indications**

These drugs achieved regulatory approval only for indications different from those tested preclinically.

| **Drug** | **Preclinical Indication** | **Approved Indication** |
| --- | --- | --- |
| 4-aminopyridine (Dalfampridine) | Spinal cord injury | Walking impairment in MS |
| Edaravone (Radicava) | Radiation/CNS injury | ALS (2017) |
| Filgrastim (G-CSF) | Myocardial infarction | Neutropenia |
| Etanercept (Enbrel) | Lung injury | Rheumatoid arthritis, psoriasis |
| Cyclosporin A | Traumatic brain injury | Transplant rejection |
| Minocycline | Spinal cord/brain injury | Bacterial infections, acne |
| Riluzole | Spinal cord injury | ALS |
| Valproate | Traumatic brain injury | Epilepsy, bipolar disorder |
| Celecoxib | Radiotherapy/skin cancer | Arthritis, pain |
| Simvastatin | Brain/CNS injury | Hypercholesterolaemia |
| Atorvastatin | Brain injury | Hypercholesterolaemia |
| Lovastatin | Radiotherapy protection | Hypercholesterolaemia |
| Memantine | Neuropathic pain | Alzheimer's disease |
| Levetiracetam (Keppra) | Head injury | Epilepsy |
| Enoxaparin | Traumatic brain injury | DVT prophylaxis |
| Mifepristone (RU38486) | PTSD | Medical abortion |
| Octreotide | Radiation enteropathy | Acromegaly, carcinoid tumours |
| Propranolol | Wound healing | Hypertension, anxiety, tremor |
| Oxandrolone | Spinal cord injury | Muscle wasting, burns |
| Sucralfate | Radiotherapy mucositis | Peptic ulcer |
| Pentoxifylline | Radiotherapy injury | Peripheral vascular disease |
| Dobutamine | Lung injury | Cardiac support |
| Melatonin | Wound healing | Sleep disorders (OTC) |
| Insulin glargine | Burns/metabolic | Diabetes mellitus |
| N-acetylcysteine | Lung/muscle injury | Paracetamol overdose, mucolytic |
| Estradiol/Oestrogen | CNS/vascular injury | Hormone replacement therapy |
| Growth hormone | Various injuries | Growth hormone deficiency |
| NNZ-2566 (Trofinetide) | Brain injury | Rett syndrome (FDA 2023) |
| Lidocaine | Neuropathic pain | Local anaesthesia |
